# Systems biology based meth-miRNA–mRNA regulatory network identifies metabolic imbalance and hyperactive cell cycle signaling involved in hepatocellular carcinoma onset and progression

**DOI:** 10.1186/s12935-019-0804-3

**Published:** 2019-04-08

**Authors:** Kashif Rafiq Zahid, Mingyang Su, Abdur Rehman Raza Khan, Shiming Han, Gou Deming, Umar Raza

**Affiliations:** 1 Shenzhen Key Laboratory of Microbial Genetic Engineering, College of Life Sciences and Oceanography, Carson International Cancer Center, Shenzhen University, Shenzhen, 518060 Guangdong China; 2 Military College of Signals, National University of Science and Technology (NUST), Khadim Hussain Rd, Rawalpindi, Pakistan; 3 School of Biological Sciences and Technology, Liupanshui Normal University, Liupanshui, 553004 China; 4 Department of Biological Sciences, National University of Medical Sciences (NUMS), Abid Majeed Road, Rawalpindi, Pakistan

**Keywords:** Systems biology, miRNA–mRNA regulatory network, Hepatocellular carcinoma, Tumor progression, Metabolic pathways, Cell cycle signaling

## Abstract

**Background:**

Hepatocellular carcinoma (HCC) is one of the leading cause of cancer associated deaths worldwide. Independent studies have proposed altered DNA methylation pattern and aberrant microRNA (miRNA) levels leading to abnormal expression of different genes as important regulators of disease onset and progression in HCC. Here, using systems biology approaches, we aimed to integrate methylation, miRNA profiling and gene expression data into a regulatory methylation-miRNA–mRNA (meth-miRNA–mRNA) network to better understand the onset and progression of the disease.

**Methods:**

Patients’ gene methylation, miRNA expression and gene expression data were retrieved from the NCBI GEO and TCGA databases. Differentially methylated genes, and differentially expressed miRNAs and genes were identified by comparing respective patients’ data using two tailed Student’s t-test. Functional annotation and pathway enrichment, miRNA–mRNA inverse pairing and gene set enrichment analyses (GSEA) were performed using DAVID, miRDIP v4.1 and GSEA tools respectively. meth-miRNA–mRNA network was constructed using Cytoscape v3.5.1. Kaplan–Meier survival analyses were performed using R script and significance was calculated by Log-rank (Mantel-Cox) test.

**Results:**

We identified differentially expressed mRNAs, miRNAs, and differentially methylated genes in HCC as compared to normal adjacent tissues by analyzing gene expression, miRNA expression, and methylation profiling data of HCC patients and integrated top miRNAs along with their mRNA targets and their methylation profile into a regulatory meth-miRNA–mRNA network using systems biology approach. Pathway enrichment analyses of identified genes revealed suppressed metabolic pathways and hyperactive cell cycle signaling as key features of HCC onset and progression which we validated in 10 different HCC patients’ datasets. Next, we confirmed the inverse correlation between gene methylation and its expression, and between miRNA and its targets’ expression in various datasets. Furthermore, we validated the clinical significance of identified methylation, miRNA and mRNA signatures by checking their association with clinical features and survival of HCC patients.

**Conclusions:**

Overall, we suggest that simultaneous (1) reversal of hyper-methylation and/or oncogenic miRNA driven suppression of genes involved in metabolic pathways, and (2) induction of hyper-methylation and/or tumor suppressor miRNA driven suppression of genes involved in cell cycle signaling have potential of inhibiting disease aggressiveness, and predicting good survival in HCC.

**Electronic supplementary material:**

The online version of this article (10.1186/s12935-019-0804-3) contains supplementary material, which is available to authorized users.

## Background

Although last two decades have witnessed a great success in prevention, diagnosis, and therapy, cancer is still one of the leading cause of death worldwide with nearly 1.6 million new cases reported only in the United States in 2016 with almost one third estimated to die of the disease [1]. Hepatocellular carcinoma (HCC) has become the most common primary hepatic malignancy, with average survival rates between 6 and 20 months [2]. Incidence rate of HCC worldwide has increased, likely due to the rising incidence of chronic hepatitis B and C infections [3]. It now ranks sixth in the world among all the malignancies, contributing to the third leading cause of mortality attributed to cancer [4]. Although recent developments in the field of functional genomics have improved our knowledge greatly, the molecular pathogenesis of HCC is not fully understood [5]. Numerous levels of transcriptional regulation are initiated during the process of tumor development and promote disease onset and progression. Hence, a better understanding of these transcriptional regulations and molecular mechanisms behind HCC onset and progression, and identification of potential biomarkers and targets are essential for effective diagnosis and therapeutic treatment.

Gene expression is regulated in many ways including promoter/gene methylation, altered transcriptional regulation, post-transcriptional modifications, mRNA transport from nucleus to cells, mRNA degradation by microRNAs (miRNAs) and post-translational modifications [6]. DNA methylation of promoter regions of tumor-suppressor genes is known to inhibit transcriptional initiation, thereby, silencing the gene expression [7]. Individual studies have shown that aberrant DNA methylation of different tumor-suppressor genes including APC [8], CDKN2A [9], RASSF1A [10] and GSTP1 [11] is correlated with clinical outcome of HCC. miRNAs are 20–22 nucleotide long, small non-coding RNAs which negatively regulate gene expression post-transcriptionally by preferentially binding to the seed sequence in the 3′-UTR of target mRNAs leading to either mRNA destabilization or degradation [12]. miRNAs are known to regulate diverse biological processes and their aberrant expression has been reported to contribute to tumorigenesis and cancer progression [13]. Aberrant expression of several miRNAs has been shown associated with disease onset, progression, therapy resistance, and metastasis in HCC [14]. Recently, whole genome profiling techniques have come up as powerful tools to identify novel molecular mechanisms and biomarkers of disease onset and progression in HCC [15], therefore, we aimed (1) to identify the pathways dysregulated in HCC due to changes in DNA methylation pattern or due to miRNA mediated post-transcriptional inhibition of genes involved by using methylation profiling, and miRNA and gene expression profiling data and (2) to construct regulatory network of disease onset and progression.

In this study, we identified differentially expressed mRNAs, miRNAs, and differentially methylated genes in HCC compared to normal adjacent tissues by analyzing gene expression, miRNA expression, and methylation profiling data of HCC patients from the National Center for Biotechnology Information Gene Expression Omnibus (NCBI GEO) and integrated top miRNAs along with mRNA targets and their methylation profile into a regulatory methylation-miRNA–mRNA (meth-miRNA–mRNA) network. Flowchart of developing this network is shown in Additional file 1: Fig. S1. Pathway enrichment analyses of identified genes revealed suppressed metabolic signaling and hyperactive cell cycle signaling as key features of HCC onset and progression which we validated in 10 different HCC patients’ datasets. Next, we confirmed the inverse correlation between gene methylation and its expression, and between miRNA and its targets’ expression in various datasets. Furthermore, we validated the clinical significance of identified methylation, miRNA, and mRNA signatures by checking their association with clinical features and survival in HCC patients.

## Methods

### Patients’ data

Patients’ gene methylation data were retrieved from the NCBI GEO database (GSE29722 [16], GSE37988 [17], GSE44909 [18], GSE54503 [19], GSE57956 [20], and GSE73003). Patients’ miRNA expression data were retrieved from the NCBI GEO database (GSE10694 [21], GSE22058 [22], GSE31384 [23], GSE36915 [24], and GSE67140). Patients’ gene expression data were retrieved from the NCBI GEO database (GSE15654 [25], GSE17856 [26], GSE20017 [27], GSE25097 [28], GSE36376 [29], GSE39791 [30], GSE45436, GSE47197, GSE55092 [31], GSE57957 [20], GSE64014, GSE76297 [32], GSE76427 [33], GSE84402 [34], GSE84598 [35], and GSE87630 [36]). In addition, gene methylation, miRNA expression and gene expression data of HCC patients at The Cancer Genome Atlas (TCGA) database were retrieved from Broad GDAC Firehose website: https://gdac.broadinstitute.org/.

### Identification of potential tumor suppressors and oncogenes in HCC using gene expression profiling data

Patients’ gene expression data retrieved from NCBI GEO database (GSE76297, GSE76427, GSE84402, and GSE84598), were normalized by calculating Z-scores for each gene within individual datasets. Later, these normalized data were pooled and gene expressions were compared between HCC and adjacent normal tissues using two tailed Student’s t-test. Bonferroni adjustment was applied to exclude false positives. Significance cut-off was taken as Bonferroni adjusted p < 0.05. Top 1000 significantly up-regulated and 1000 down-regulated genes in HCC as compared to adjacent normal tissues were selected and their potential to predict overall survival in HCC patients from TCGA database was checked to identify potential tumor suppressors and oncogenes. Schematic workflow is shown in Additional file 1: Fig. S1. Overall, the genes which were significantly down-regulated in HCC tumor tissues compared to adjacent normal tissues and whose higher expression was associated with good survival in HCC patients were selected as potential tumor suppressors (n = 335), and the genes which were significantly up-regulated in HCC tumor tissues compared to adjacent normal tissues and whose higher expression was associated with poor survival in HCC patients were selected as potential oncogenes (n = 415) (Additional file 2: Table S1).

### Functional annotation and enrichment analysis

**Fig. 1:**
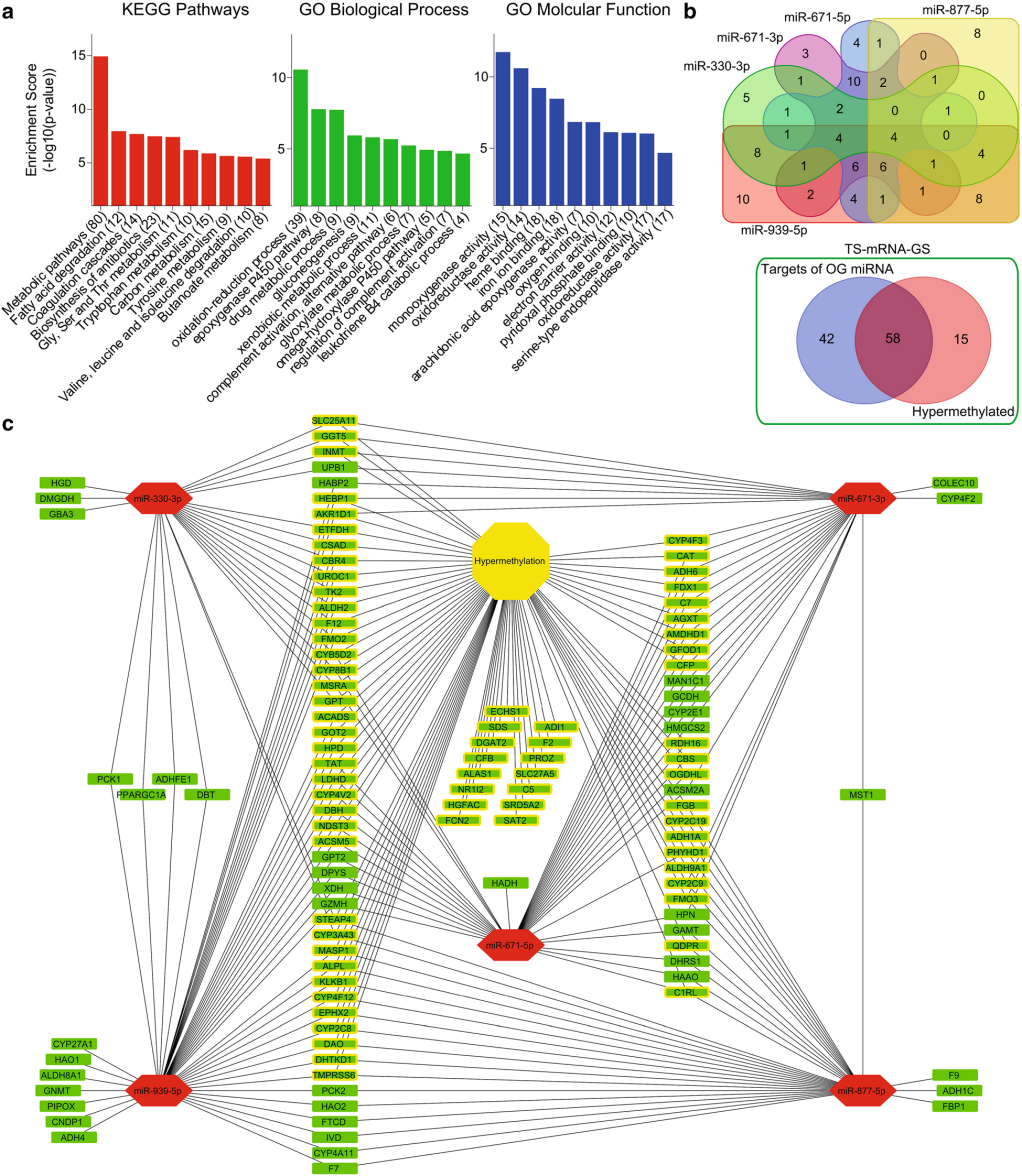
Metabolic signaling enriched meth-miRNA–mRNA network of tumor suppressor genes in HCC. Graphs showing top 10 KEGG pathways and functional annotation GO terms associated with potential tumor suppressors (**a**). Venn diagrams showing number of shared tumor suppressor targets of top 5 oncogenic miRNAs (top) and number of shared tumor suppressors whose expression can either be modulated by gene hypermethylation and/or due to targeting by oncogenic miRNAs in TS-mRNA-GS (bottom) (**b**). meth-miRNA–mRNA network showing tumor suppressors being targeted by oncogenic miRNAs. Rectangles indicate genes/mRNAs and hexagons indicate miRNAs. Red represents high expression and green represents low expression in HCC as compared to adjacent normal tissues. Genes whose expressions are found to be down-regulated due to gene hyper-methylation in HCC are shown with yellow border (**c**)

**Fig. 2:**
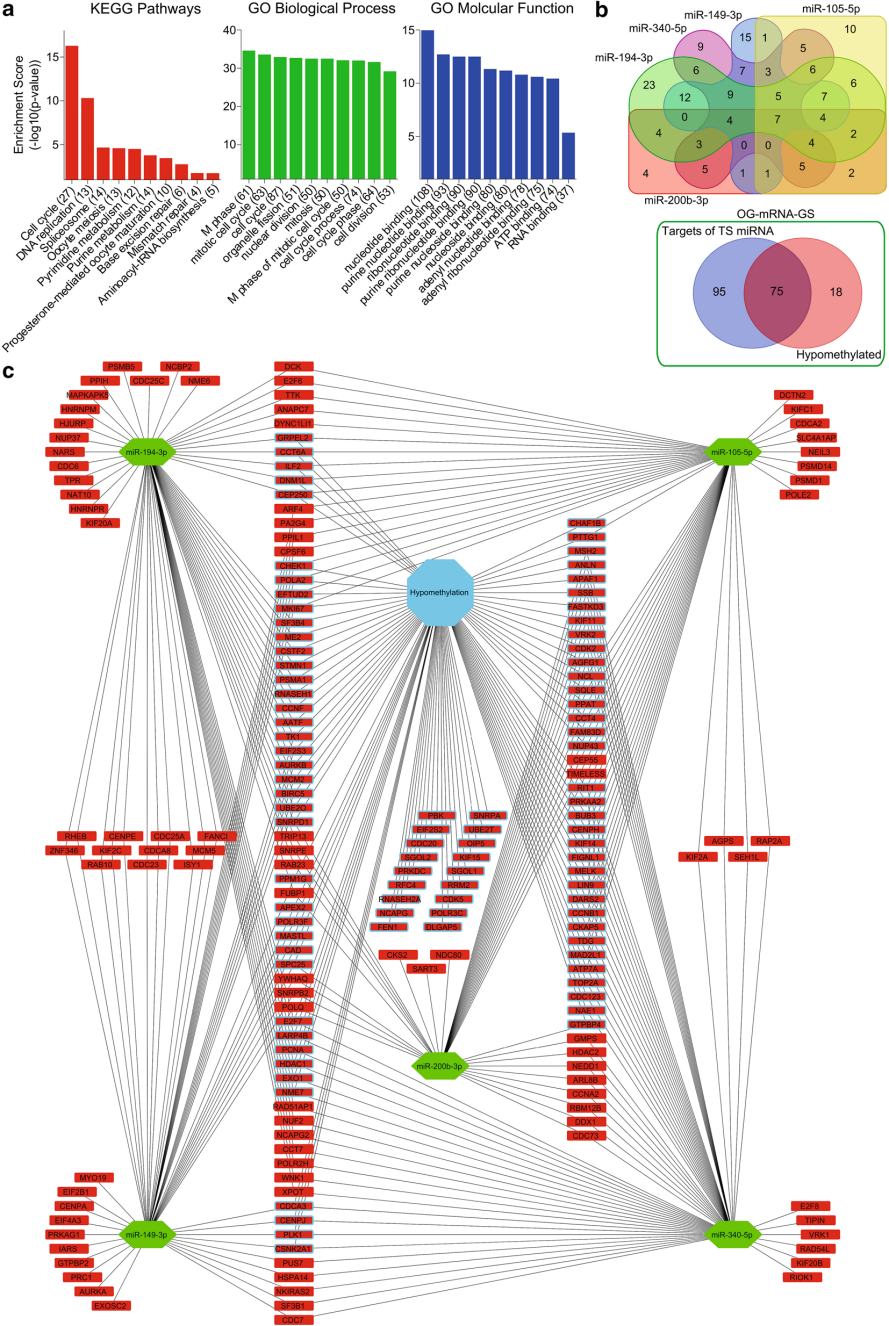
Cell cycle signaling enriched meth-miRNA–mRNA network of oncogenes in HCC. Graphs showing top 10 KEGG pathways and functional annotation GO terms associated with potential oncogenes (**a**). Venn diagrams showing number of shared oncogenic targets of top 5 tumor suppressor miRNAs (top) and number of shared oncogenes whose expression can either be modulated by gene hypo-methylation and/or due to targeting by tumor suppressor miRNAs in OG-mRNA-GS (bottom) (**b**). meth-miRNA–mRNA network showing oncogenes being targeted by tumor suppressor miRNAs. Rectangles indicate genes/mRNAs and hexagons indicate miRNAs. Red represents high expression and green represents low expression in HCC as compared to adjacent normal tissues. Genes whose expressions are found to be up-regulated due to gene hypo-methylation in HCC are shown with blue border (**c**)

Lists of potential tumor suppressors and oncogenes were separately uploaded to online freely available DAVID functional annotation tool (https://david.ncifcrf.gov/summary.jsp) to identify GO biological process, GO molecular function, and KEGG pathways suppressed or hyperactive in HCC as compared to adjacent normal tissues. Tumor suppressors (n = 133) and oncogenes (n = 211) were identified by combining top 10 functional annotations from individual analysis (Figs. 1a and 2a).

### Identification of differentially expressed catabolic and anabolic enzymes in HCC

Gene lists associated with top deregulated pathways like fatty acid degradation, amino acid metabolism, and purine and pyrimidine metabolism etc. were downloaded from online available KEGG database and were combined in one list. This list was then compared with the pool of previously identified top 1000 significantly up and down regulated genes in HCC as compared to normal tissues to identify the differentially expressed genes. Next, a total of 109 genes were shortlisted as having enzymatic function among differentially expressed genes. Later, web and literature based search was done to identify the type of function (anabolic/catabolic) for these enzymes. Overall, this analysis showed that out of 39 anabolic enzymes, 31 were upregulated and 8 were downregulated. On the other hand, out of 70 catabolic enzymes, 68 were downregulated and only 2 were upregulated. Cumulative catabolic and anabolic signature scores were calculated by adding the average Z-score of all the catabolic and anabolic enzymes respectively for both HCC and adjacent normal tissues from meta-analysis of data retrieved from NCBI GEO database (GSE76297, GSE76427, GSE84402, and GSE84598) (Additional file 3: Fig. S2).

### Identification of potential tumor suppressor and oncogenic miRNAs in HCC using miRNA expression profiling data

Patients’ miRNA expression data were retrieved from NCBI GEO database (GSE10694, GSE22058, GSE31384, and GSE36915). miRNA expressions were compared between HCC and adjacent normal tissues using two tailed Student’s t-test to identify differentially expressed miRNAs. Bonferroni adjustment was applied to exclude false positives. Significance cut-off was taken as Bonferroni adjusted p < 0.05. All the significantly up-regulated and down-regulated miRNAs in HCC as compared to adjacent normal tissues were selected and their potential to predict overall survival in HCC patients from GEO dataset GSE31384 was checked to identify potential tumor suppressors and oncogenic miRNAs. Schematic workflow is shown in Additional file 1: Fig. S1. Overall, the miRNAs which were significantly down-regulated in HCC tumor tissues compared to adjacent normal tissues and whose higher expression was associated with good survival in HCC patients were selected as potential tumor suppressor miRNAs (n = 46), and the genes which were significantly up-regulated in HCC tumor tissues compared to adjacent normal tissues and whose higher expression was associated with poor survival in HCC patients were selected as potential oncogenic miRNAs (n = 27) (Additional file 4: Table S2).

### Identification of differentially methylated genes in HCC using methylation profiling data

Patients’ methylation expression data were retrieved from NCBI GEO database (GSE29722, GSE37988, GSE44909, GSE54503, GSE57956, and GSE73003). Methylation scores were compared between HCC and adjacent normal tissues using two tailed Student’s t-test with significance cut-off of p < 0.05 to identify differentially methylated genes in HCC; hyper methylated (n = 2399) and Hypomethylated (n = 1243).

### miRNA–mRNA inverse pairing using miRDIP tool

Lists of identified tumor suppressor miRNAs (n = 46) and oncogenes (n = 211) were uploaded to online freely available tool miRDIP v4.1 (http://ophid.utoronto.ca/mirDIP/) to identify the oncogenic targets of tumor suppressor miRNAs. Top 5 tumor suppressor miRNAs with maximum targets among oncogenes were selected to be included in the network (Additional file 5: Table S3 and Fig. 2b; Top panel). Similarly, lists of identified oncogenic miRNAs (n = 27) and tumor suppressors (n = 133) were uploaded to the same tool to identify the tumor suppressor targets of oncogenic miRNAs. Top 5 oncogenic miRNAs with maximum targets among tumor suppressors were selected to be included in the network (Additional file 5: Table S3 and Fig. 1b; Top panel).

### Identification of hypermethylated tumor suppressors and hypomethylated oncogenes

List of tumor suppressors was compared with that of hypermethylated genes to identify tumor suppressors which can be down-regulated due to gene hyper-methylation in HCC tissues as compared to adjacent normal tissues (n = 73) (Additional file 6: Table S4). Similarly, list of oncogenes was compared with that of hypomethylated genes to identify oncogenes which can be up-regulated due to gene hypo-methylation in HCC tissues as compared to adjacent normal tissues (n = 93) (Additional file 6: Table S4).

### Constructing meth-miRNA–mRNA network

miRNA-target interaction and methylation profiles were uploaded to Cytoscape software v3.5.1 to create tumor suppressor and oncogenic meth-miRNA–mRNA networks (Figs. 1c and 2c). In case, an mRNA was a target of more than 2 miRNAs, only top two miRNA–mRNA interactions were shown based on confidence score from miRDIP4 tool to keep interactions visually distinguishable in the network. Genes whose expression is predicted to change due to changing methylation pattern are marked by border of different color.

### Gene sets, gene signatures, and Z-score calculation

Gene sets related to metabolic processes and cell cycle signaling were downloaded from the GSEA website: http://software.broadinstitute.org/gsea/index.jsp. To calculate the scores for these gene sets, Z-scores of all the mRNAs in the given GS were summed for each patient. Two gene signatures (GS), consisting of list of genes identified as hypermethylated tumor suppressors and hypomethylated oncogenes in the meth-miRNA–mRNA network were named as hypermethylated-GS and hypomethylated-GS respectively. To calculate the scores for individual GS, methylation score of all the gene in the given GS were summed for each patient whereas to calculate the scores for methylation-GS (meth-GS), hypomethylated-GS score was subtracted from hypermethylated-GS score for each patient [37]. This implies that the patients having high meth-GS score will have higher methylation of tumor suppressor genes than oncogenes and vice versa. Two GS, consisting of list of miRNAs identified as tumor suppressors or oncogenic ones were named as TS-miRNA-GS and OG-miRNA-GS respectively. To calculate the scores for individual GS, Z-scores of all the miRNAs in the given GS were summed for each patient whereas to calculate the scores for miRNA-GS, OG-miRNA-GS score was subtracted from TS-miRNA-GS score for each patient [37]. This implies that the patients having high miRNA-GS score will have higher expression of tumor suppressor miRNAs than oncogenic miRNAs and vice versa. Two GS, consisting of list of mRNAs identified as tumor suppressors or oncogenes were named as TS-mRNA-GS (Fig. 1b, Bottom panel) and OG-mRNA-GS (Fig. 2b, Bottom panel) respectively. To calculate the scores for individual GS, Z-scores of all the mRNAs in the given GS were summed for each patient whereas to calculate the scores for mRNA-GS, OG-mRNA-GS score was subtracted from TS-mRNA-GS score for each patient [37]. This implies that the patients having high mRNA-GS score will have higher expression of tumor suppressors than oncogenes and vice versa.

### Gene Set Enrichment Analysis (GSEA)

GSEA was performed using gene sets related to tumor progression, proliferation, and survival in HCC downloaded from the GSEA website: http://software.broadinstitute.org/gsea/index.jsp. Significance cut-off was taken as nominal p < 0.05.

### Survival analysis

Survival analyses for all the genes in TCGA database and for all the miRNAs in GEO dataset GSE31384 were performed using R script [38]. Survival curves were generated using Kaplan–Meier method. Patients without any available survival time or event were excluded from the corresponding patient groups. All the separations were done from the median. Significance of the differences in survival between two groups was calculated by Log-rank (Mantel-Cox) test. Significance cut-off was taken as p < 0.05.

### Statistical analyses

Venn diagrams were made using online freely available tool at http://bioinformatics.psb.ugent.be/webtools/Venn/. For correlation analyses, Pearson correlation co-efficients were calculated. Comparisons between two groups were made by two tailed Student’s t-test. Significance cut-off for correlation analyses and Student’s t-test was taken as p < 0.05.

## Results

### Constructing meth-miRNA–mRNA networks suggested suppressed metabolic signaling and hyperactive cell cycle signaling in HCC

In order to identify protein coding players of tumor progression in HCC, we performed a meta-analysis using expression profiling data of HCC tumors and their adjacent normal tissues from 4 different online available GEO patients’ datasets (Additional file 1: Fig. S1; For details, see “Methods” section) and identified significantly up- and down-regulated genes in HCC tumor tissues as compared to adjacent normal tissues. Next, we narrowed down this list of genes by applying a stringent survival analysis threshold where we tested whether differential expression of these genes is associated with survival of HCC patients from TCGA database. Overall, theses analyses identified 335 potential tumor suppressor genes which were down-regulated in HCC tumor tissues compared to normal tissues and whose higher expression was associated with good survival in HCC patients, and 415 potential oncogenes which were up-regulated in HCC tumor tissues compared to normal tissues and whose higher expression was associated with poor survival in HCC patients (Additional file 2: Table S1). Functional annotation and pathway enrichment analyses of potential tumor suppressor genes suggested that metabolic pathways are suppressed in HCC as compared to adjacent normal liver tissues (Fig. 1a). On the other hand, functional annotation and pathway enrichment analyses of potential oncogenes suggested that cell cycle signaling is hyperactive in HCC as compared to adjacent normal liver tissues (Fig. 2a). As major upregulated and downregulated pathways like fatty acid degradation, amino acid metabolism, and purine and pyrimidine metabolism etc. (Figs. 1a and 2a) involved both anabolic and catabolic enzymes, we compared catabolic and anabolic signature scores between tumor and normal liver tissues. Notably, anabolic enzymes were mainly upregulated whereas catabolic enzymes were mainly downregulated in HCC as compared to adjacent normal tissues (Additional file 3: Fig. S2; For details, see “Methods” section) further confirming that metabolic imbalance is a key feature in regulating HCC onset and progression.

Next, to identify miRNA modulators of tumor progression in HCC, we performed miRNA expression profiling analysis using data of HCC tumors and their adjacent normal tissues from four different online available GEO patients’ datasets (Additional file 1: Fig. S1; for details, see “Methods” section) and identified significantly up- and down-regulated miRNAs in HCC tumor tissues as compared to adjacent normal tissues. Next, we narrowed down this list of genes by applying a stringent survival analysis threshold where we tested whether differential expression of these miRNAs is associated with survival of HCC patients from GEO patient’s dataset GSE31384 (Additional file 4: Table S2). Overall, theses analyses identified 46 potential tumor suppressor miRNAs which were down-regulated in HCC tumor tissues compared to normal tissues and whose higher expression was associated with good survival in HCC patients, and 29 potential oncogenic miRNAs which were up-regulated in HCC tumor tissues compared to normal tissues and whose higher expression was associated with poor survival in HCC patients. Later, we performed miRNA–mRNA inverse pairing analysis and identified oncogenic targets of tumor suppressor miRNAs and tumor suppressor targets of oncogenic miRNAs. Top 5 oncogenic and tumor suppressor miRNAs having maximum targets (Additional file 4: Table S2, Figs. 1b; top panel and Fig. 2b; top panel) were incorporated into tumor suppressor and oncogenic miRNA–mRNA networks, respectively, along with their targets (Figs. 1c and 2c).

Next, we performed methylation profiling analysis using data of HCC tumors and their adjacent normal tissues from six different online available GEO patients’ datasets (Additional file 1: Fig. S1; for details, see “Methods” section) and identified significantly hyper- and hypomethylated genes in HCC tumor tissues as compared to adjacent normal tissues. Comparing these lists of genes with those of potential tumor suppressor and oncogenes helped us in identifying hypermethylated tumor suppressors and hypomethylated oncogenes (Additional file 5: Table S3). Interestingly, most of tumor suppressors and oncogenes affected by hyper- and hypo-methylation were also targets of oncogenic and tumor suppressor miRNAs respectively (Figs. 1b; bottom panel and 2b; bottom panel). Lastly, methylation profiles of tumor suppressors and oncogenes were incorporated into respective miRNA–mRNA networks to develop tumor suppressor and oncogenic meth-miRNA–mRNA networks respectively (Figs. 1c and 2c).

Overall, we constructed a tumor suppressor and an oncogenic network. The former comprises of oncogenic miRNAs targeting tumor suppressor genes involved in metabolic signaling, whose expression can also be suppressed due to hyper-methylation in HCC as compared to adjacent normal tissues. Whereas, the latter comprises of tumor suppressor miRNAs targeting oncogenes involved in cell cycle signaling, which can also be overexpressed due to hypo-methylation in HCC as compared to adjacent normal tissues.

### Metabolic imbalance and hyperactive cell cycle signaling are key features of HCC onset and progression

**Fig. 3:**
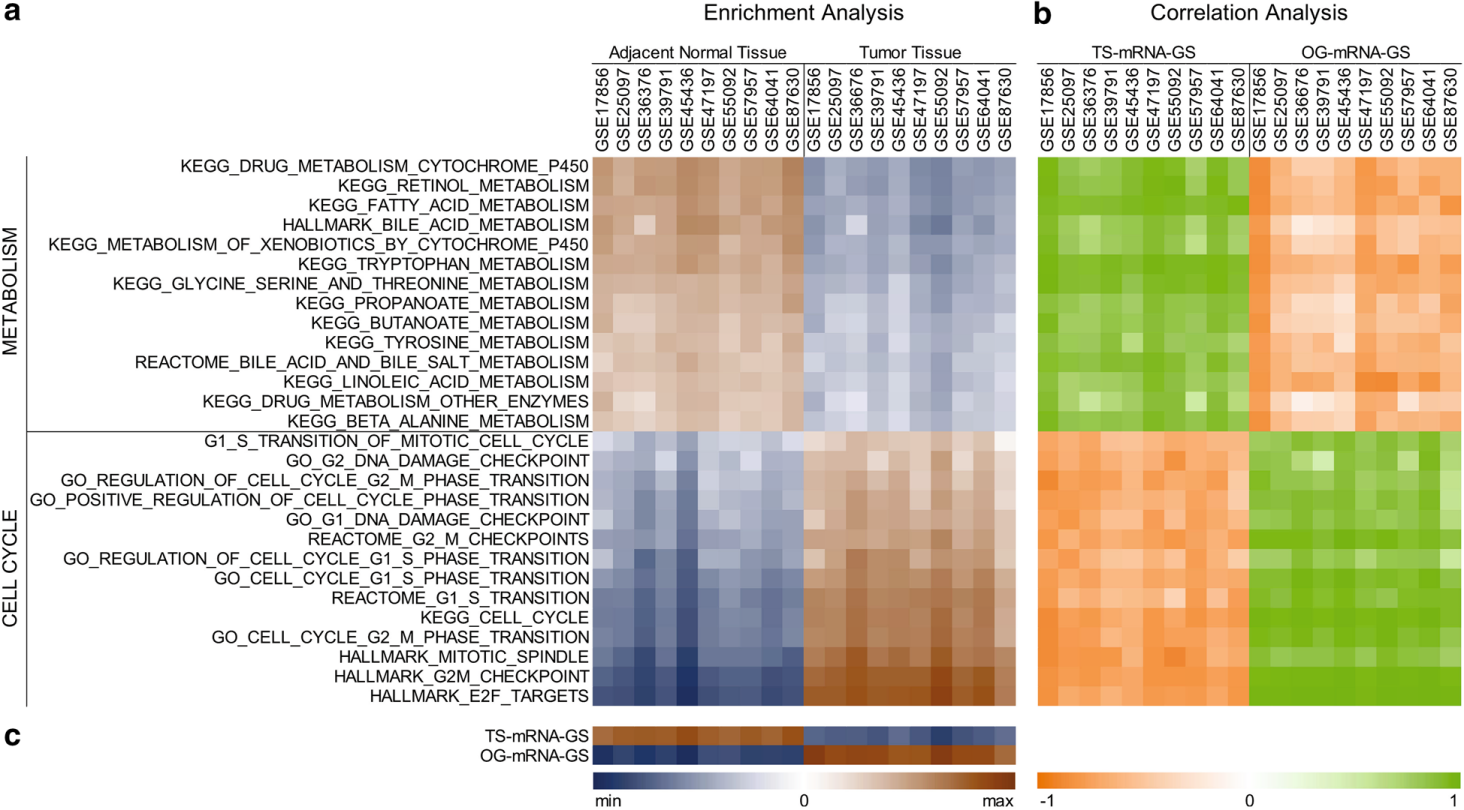
Metabolic imbalance and hyperactive cell cycle signaling are key features of HCC onset and progression. Heatmap showing enrichment of already published metabolic pathways associated gene sets (n = 14) and cell cycle signaling associated gene sets (n = 14) in HCC tissues and adjacent normal tissues of patients from 10 different GEO datasets (**a**). Heatmap showing correlation analyses of Z-scores of already published metabolic pathways associated gene sets (n = 14) and cell cycle signaling associated gene sets (n = 14) with our TS-mRNA-GS and OG-mRNA-GS scores (**b**). Heatmap showing enrichment of TS-mRNA-GS and OG-mRNA-GS in HCC tissues and adjacent normal tissues of patients from 10 different GEO datasets (**c**)

As functional annotation and pathway enrichment analyses revealed that metabolic pathways are suppressed whereas cell cycle signaling is hyperactive in HCC compared to adjacent normal tissues, we aimed to validate our findings. In this line, we calculated the Z-score of different gene sets associated with metabolic pathways and cell cycle signaling for the patients from 10 different GEO datasets and compared them between HCC and adjacent normal tissues. Gene sets associated with metabolic pathways were significantly suppressed in HCC compared to adjacent normal tissues whereas gene sets associated with cell cycle signaling were significantly enriched in HCC compared to adjacent normal tissues confirming that metabolic imbalance and hyperactive cell cycle signaling are key features of HCC onset and progression (Fig. 3a). Interestingly, all the gene sets associated with metabolic pathways were positively correlated with our TS-mRNA-GS and those associated with cell cycle signaling were positively correlated with our OG-mRNA-GS (Fig. 3b). Notably, we observed highest difference between HCC and adjacent normal tissue in terms of TS-mRNA-GS and OG-mRNA-GS scores (Fig. 3c) as compared to any other gene set associated with metabolic pathways or cell cycle signaling (Fig. 3a) suggesting that the gene signatures (TS-mRNA-GS and OG-mRNA-GS) we developed have greater sensitivity and specificity to identify HCC onset and progression.

### Hyper-methylation driven inhibition of metabolic pathways and hypo-methylation driven activation of cell cycle signaling are associated with aggressive state and poor survival in HCC

**Fig. 4:**
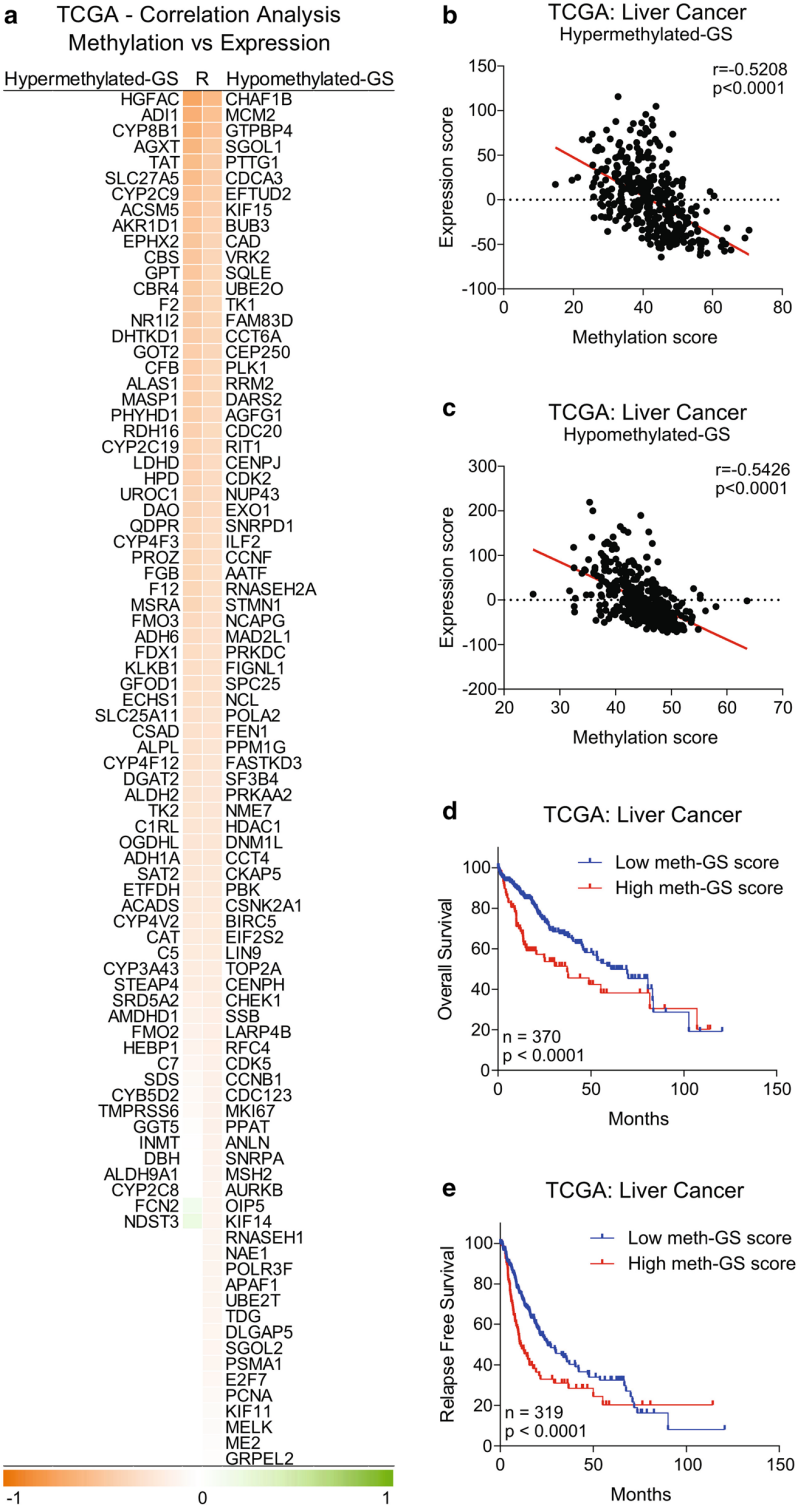
Hyper-methylation driven inhibition of metabolic pathways and hypo-methylation driven activation of cell cycle signaling are associated with poor survival in HCC. Heatmap showing correlation analysis between methylation status and mRNA expression of genes identified as hyper- and hypomethylated in HCC as compared to adjacent normal tissues. Both lists, hypermethylated-GS and hypomethylated-GS are sorted in ascending order based on correlation coefficient R (**a**). Scatter-plot showing correlation analysis between methylation score and expression score of hypermethylated-GS (**b**) and hypomethylated-GS (**c**) in HCC patients from TCGA database (n = 370). Kaplan–Meier survival plots representing the percentage overall survival in HCC patients from TCGA database (n = 370) based on low vs high meth-GS score (**d**). Kaplan–Meier survival plots representing the percentage relapse free survival in HCC patients from TCGA database (n = 319) based on low vs high meth-GS score (**e**)

After establishing that suppressed metabolic processes and hyperactive cell cycle signaling are major features of HCC onset and progression, we aimed to validate the methylation mediated regulation of metabolism and cell cycle signaling in HCC as proposed in the meth-miRNA–mRNA network and to identify its clinical significance in HCC. To this end, first of all, we checked the correlation of methylation status of genes we identified as hypermethylated or hypomethylated in HCC with their mRNA expression in patients from TCGA database and found an inverse correlation between methylation and expression of these genes (Fig. 4a–c) suggesting that methylation profile proposed in meth-miRNA–mRNA network has potential to regulate the metabolic pathways and cell cycle signaling in HCC. Next, we calculated meth-GS scores by subtracting the hypomethylated-GS score from hypermethylated-GS score for each patient (For details, see “Methods” section) and checked whether it is associated with clinical features and survival of HCC patients from TCGA database. Vascular invasion is a critical parameter of tumor progression and disease aggressiveness in HCC [39]. We observed that metabolic pathways associated genes are more methylated than cell cycle signaling associated genes in patients who experienced vascular invasion as they had high meth-GS score as compared to those who have no signs of vascular invasion (Additional file 7: Fig. S3a). We also observed high meth-GS score in stage III patients, Grade 3 tumors and in pathological tumor size T3 as compared to in stage I patient, Grade 1 tumors and in pathological tumor size T1 respectively (Additional file 7: Fig. S3b–d). Notably, we found that high meth-GS score is associated with poor overall survival and relapse free survival in HCC patients from TCGA database (Fig. 4d, e) confirming that hyper-methylation driven inhibition of metabolic pathways and hypo-methylation driven activation of cell cycle signaling are associated with aggressive disease state and poor survival in HCC.

### miRNA-GS is associated with less aggressive state and good survival in HCC patients

**Fig. 5:**
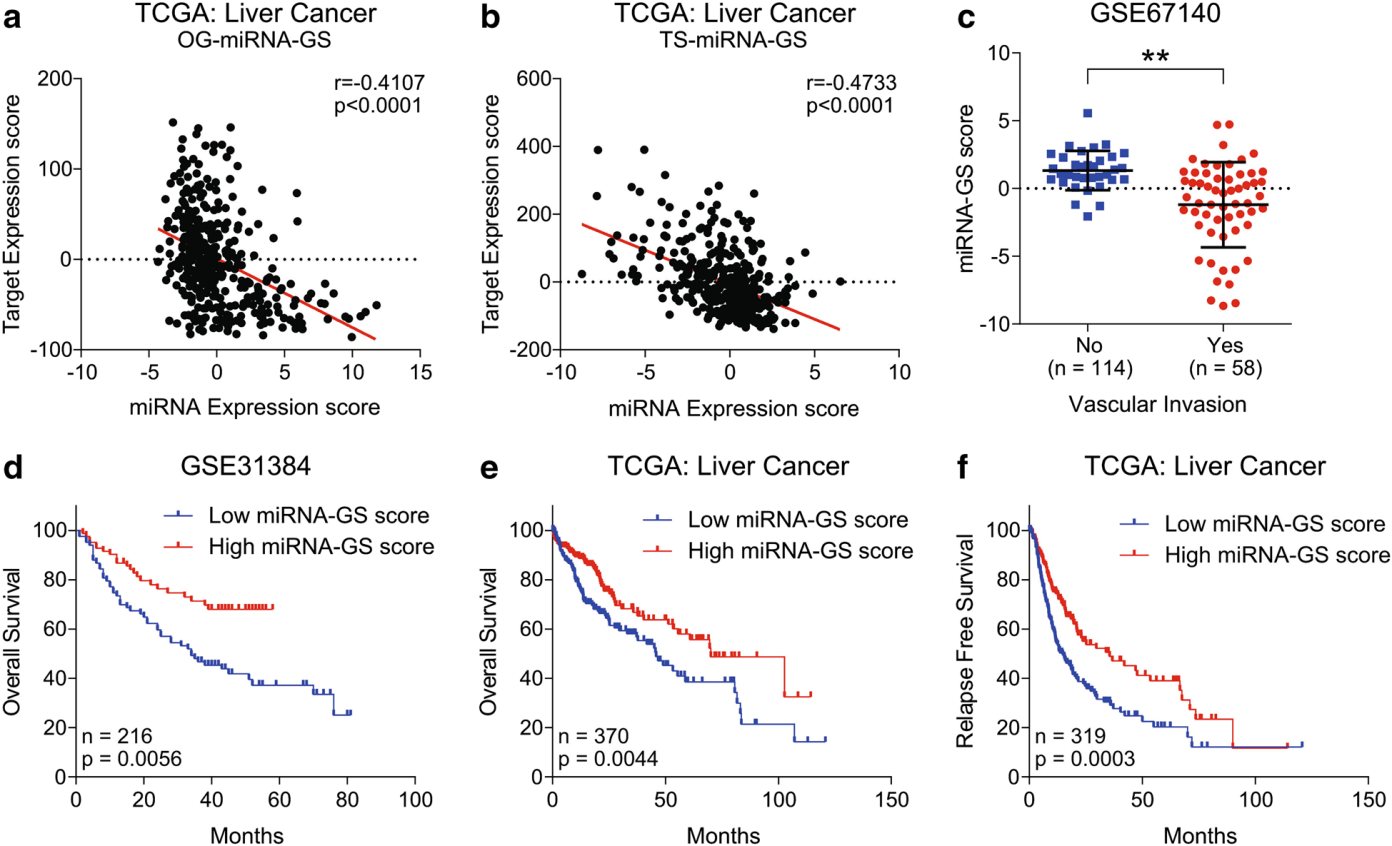
miRNA-GS is associated with less aggressive state and good survival in HCC patients. Scatter-plot showing correlation analysis between miRNA expression score and their targets’ expression score for OG-miRNA-GS (**a**) and TS-miRNA-GS (**b**) in HCC patients from TCGA database (n = 368). Dot-plot showing miRNA-GS-score in HCC patients who experienced or did not experience vascular invasion in GEO dataset GSE67140 (**c**). Kaplan–Meier survival plots representing the percentage overall survival in HCC patients from GSE31384 (n = 216) (**d**) and from TCGA database (n = 370) (**e**) based on low vs high miRNA-GS score. Kaplan–Meier survival plots representing the percentage relapse free survival in HCC patients from and TCGA database (n = 319) based on low vs high miRNA-GS score (**f**). *p < 0.05; **p < 0.01; ns, not significant, Student’s t-test

In order to validate the miRNA mediated regulation of metabolism and cell cycle signaling in HCC as proposed in the meth-miRNA–mRNA network and to identify its clinical significance in HCC, we first checked the correlation of expression of oncogenic and tumor suppressor miRNAs with their tumor suppressor and oncogenic targets respectively (Fig. 5a, b) and found inverse correlations suggesting that miRNA–mRNA interactions proposed in meth-miRNA–mRNA network also have potential to regulate the metabolic pathways and cell cycle signaling in HCC. Next, we calculated miRNA-GS scores by subtracting the OG-miRNA-GS score from TS-miRNA-GS score for each patient (For details, see “Methods” section) and checked whether it is associated with clinical features and survival of HCC patients. We observed that suppression of metabolic pathways associated genes by oncogenic miRNA is stronger than suppression of cell cycle signaling associated genes by tumor suppressor miRNAs in patients who experienced vascular invasion as they had low miRNA-GS score as compared to those who have no signs of vascular invasion (Fig. 5c). Notably, we found that high miRNA-GS score is also associated with good overall survival and relapse free survival in HCC patients from GEO dataset GSE31384 and TCGA database (Fig. 5d–f) confirming that reversal of oncogenic miRNA mediated suppression of metabolic pathways and induction of tumor suppressor miRNA mediated suppression of cell cycle signaling would lead to a less aggressive disease state and good survival in HCC.

### mRNA-GS is associated with less aggressive state and good survival in HCC patients

**Fig. 6:**
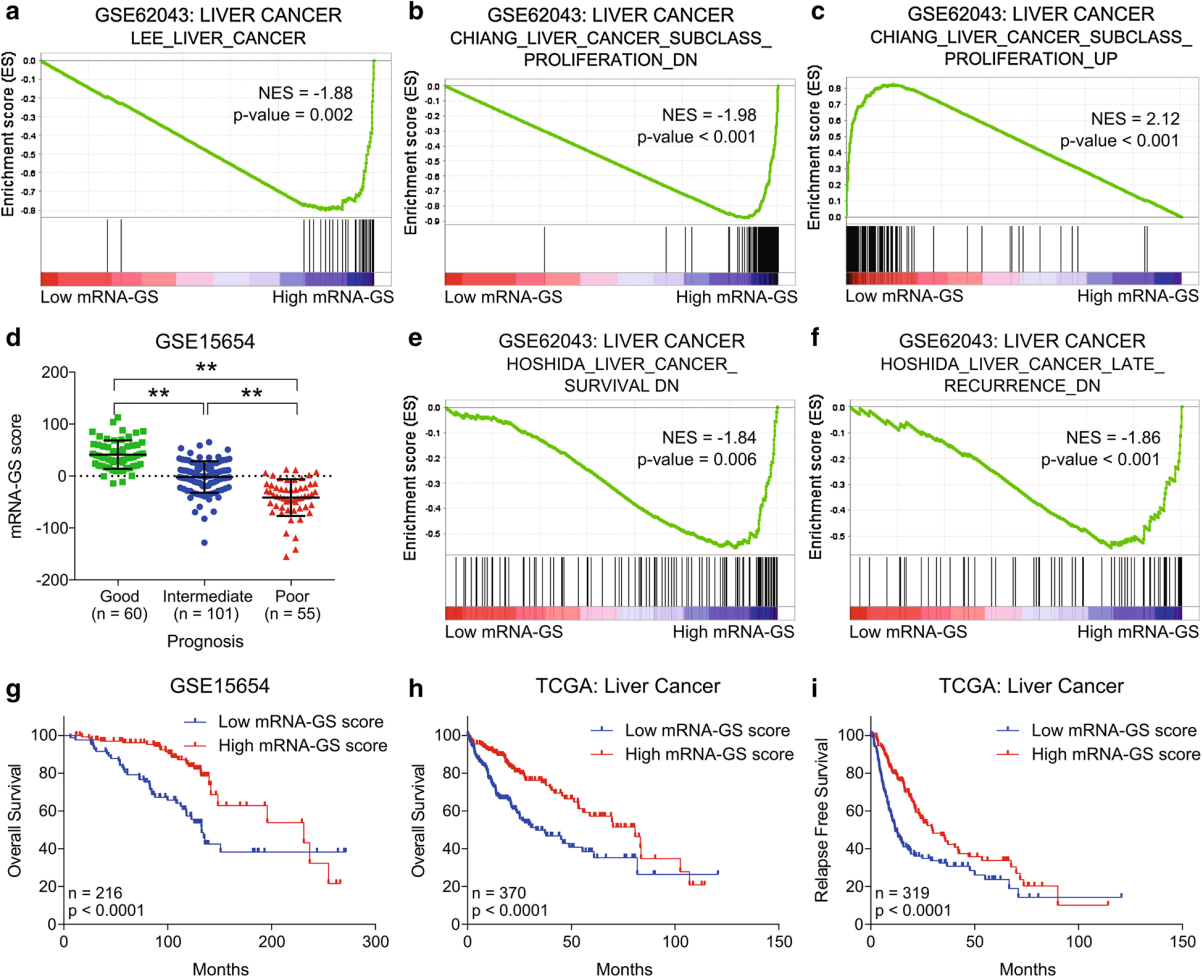
mRNA-GS is associated with less aggressive state and good survival in HCC patients. Gene set enrichment plots of HCC patients from GSE62043 (n = 100) with low or high mRNA-GS score. Genes down-regulated in tumor compared to non-tumor liver samples from patients with HCC (**a**) and top 200 marker genes down-regulated in the ‘proliferation’ subclass of HCC; characterized by increased proliferation, high levels of serum AFP, and chromosomal instability, are enriched in patients having high mRNA-GS scores (**b**) whereas top 200 marker genes up-regulated in the ‘proliferation’ subclass of HCC are enriched in patients having low mRNA-GS score (**c**). Dot-plot showing mRNA-GS scores in HCC patients having good, intermediate or poor prognosis in GEO dataset GSE15654 (**d**). Gene set enrichment plots of HCC patients from GSE62043 (n = 100) with low or high mRNA-GS score. Genes whose expressions are correlated with good survival of HCC patients (**e**) and genes whose expressions are correlated with lower risk of late recurrence in HCC (**f**) are enriched in patients having high mRNA-GS score. Kaplan–Meier survival plots representing the percentage overall survival in HCC patients from GSE15654 (n = 216) (**g**) and from TCGA database (n = 370) (**h**) based on low vs high mRNA-GS score. Kaplan–Meier survival plots representing the percentage relapse free survival in HCC patients from and TCGA database (n = 319) based on low vs high mRNA-GS score (**i**)

In order to validate the clinical significance of protein coding genes in meth-miRNA–mRNA networks, we calculated mRNA-GS scores by subtracting the OG-mRNA-GS score from TS-mRNA-GS score for each patient (For details, see “Methods” section), separated the patients’ data into low and high mRNA-GS expression groups, and performed Gene Set Enrichment Analysis (GSEA). The results showed that genes down-regulated in HCC tumor tissues as compared to non-tumor tissues are enriched in patients having high mRNA-GS scores (Fig. 6a) confirming that the proposed TS-mRNA-GS is down-regulated whereas the OG-mRNA-GS is up-regulated in HCC tissues. In addition, the genes down-regulated in “proliferation” subclass of liver cancer were enriched in patients having high mRNA-GS score whereas genes up-regulated in “proliferation” subclass of liver cancer were enriched in patients having low mRNA-GS score (Fig. 6b, c) suggesting that the proposed imbalance of metabolic and cell cycle signaling can lead to increase in proliferative potential of HCC tumor cells. mRNA-GS is found positively correlated with prognosis in HCC as mRNA-GS score is higher in patients having good prognosis as compared to those having intermediate or poor prognosis (Fig. 6d). In two separate patient datasets, we observed that mRNA-GS was significantly down-regulated in patients who experienced vascular invasion as compared to those having no signs of vascular invasion (Additional file 8: Fig. S4a, b). We also observed high mRNA-GS score in stage III patients, Grade 3 tumors and in pathological tumor size T3 as compared to in stage I patient, Grade 1 tumors and in pathological tumor size T1 respectively (Additional file 8: Fig. S4c–e) confirming that the suppression of metabolic pathways and hyperactivation of cell cycle signaling are associated with aggressive and advanced disease state in HCC. GSEA revealed that genes whose downregulation is associated with poor survival in HCC and genes whose expression is correlated with lower risk of late recurrence in HCC are enriched in HCC patients having high mRNA-GS scores (Fig. 6e, f). Notably, we found that high mRNA-GS score is also associated with good overall survival and relapse free survival in HCC patients from GEO dataset GSE15654 and TCGA database (Fig. 6g–i) confirming that restoring metabolic pathways and suppressing cell cycle signaling would lead to a less aggressive disease state and good survival in HCC.

## Discussion

In this study, we systematically identified differentially expressed mRNAs, miRNAs, and differentially methylated genes in HCC compared to normal adjacent tissues by analyzing gene expression, miRNA expression, and methylation profiling data of HCC patients from NCBI GEO database and integrated top miRNAs along with mRNA targets and their methylation profile into a regulatory meth-miRNA–mRNA network (Figs. 1 and 2). Changes in DNA and/or promoter methylation patterns of individual or set of genes have been implicated in HCC onset and progression by modulating gene expression patterns of individual cells and/or tissue as a whole [8–11, 18], but a systematic methylation signature based study was lacking in the field. To the best of our knowledge, this is the first instance where altered methylation profile between tumor and adjacent normal tissues has been integrated into a network along with expression profiling data in HCC. We found inverse correlation between gene methylation and its expression for most of the identified hypo- and hyper-methylated genes (Fig. 4a–c) which needs to be validated in vitro and in vivo to certify the identified meth-GS as prognostic factor for clinical outcome and survival in HCC patients (Fig. 4d, e, Additional file 7: Fig. S3).

Similar to changes in DNA methylation pattern, altered miRNA expression has also been shown to regulate gene expression, but at post-transcriptional level [13, 40]. Considerable amount of research has identified differentially expressed miRNAs between HCC and adjacent normal tissues; thus, regulating cellular processes like metabolism, cell cycle, proliferation and apoptosis by suppressing the expression of their target mRNAs [41]. Most of these studies were based on single miRNA/single target approach and very few studies have endeavored to identify the combinatorial effect of differentially expressed miRNAs in HCC by miRNA-gene interaction networks [42, 43]. In this study, we have reported five oncogenic miRNAs (miR-330-3p, miR-671-3p, miR-671-5p, miR-877-5p and miR-939-5p) which are upregulated in HCC, and five tumor suppressor miRNAs (miR-105-5p, miR-149-3p, miR-194-3p, miR-200b-3p and miR-340-5p) which tend to be downregulated in HCC (Figs. 1 and 2). Notably, some of these miRNAs have already been proposed as oncogenic or tumor suppressor miRNAs in HCC or other cancer types confirming the significance of our findings. For instance, miR-330-3p has been reported as oncogenic miRNA in non-small-cell cancer promoting cell proliferation, cell invasion and metastasis [44, 45] whereas miR-105-5p was found downregulated in HCC and correlated with good prognosis and survival in HCC [46]. In addition, miR-149-3p has been shown inhibiting proliferation, migration and invasion in bladder cancer [47] and miR-200b-3p was found to inhibit growth and metastasis in breast and prostate cancer cells by regulating different targets [48, 49]. As aberrant miRNA expression go hand in hand with altered methylation pattern to modulate gene expression patterns and regulating specific oncogenic phenotype, we integrated both parameters along with gene expression patterns into our regulatory meth-miRNA–mRNA network to better understand the molecular basis of HCC onset and progression. We found inverse correlations between expression of miRNAs and their targets (Fig. 5a, b) which needs to be validated in vitro and in vivo to certify the identified miRNA-GS as diagnostic factor for clinical outcome and survival in HCC patients (Fig. 5c–f).

Pathway enrichment analyses of identified tumor suppressors and oncogenes revealed suppression of metabolic pathways like fatty acid degradation and amino acid metabolism, and hyperactive cell cycle signaling assisted with aberrant DNA replication and, purine and pyrimidine metabolism as key features of HCC onset and progression (Figs. 1 and 2) which we validated in 10 different HCC patients’ datasets (Fig. 3) and found correlated with clinical characteristics and survival in HCC patients (Fig. 6d–i, Additional file 8: Fig. S4). Liver is metabolic hub of the body and plays crucial part in lipid and fatty acid metabolism. Increasing evidence has shown that aberrant activation of lipogenesis due to deregulated lipid metabolism play key role in regulating tumor growth and metastasis in HCC [50–52]. Progression through cell cycle is tightly controlled by cyclins and cyclin-dependent kinases (CDKs) and aberrant expression of these genes can trigger cell cycle programming to run at a faster pace [53]. We observed upregulation of different cyclins (CCNA2, CCNB1, CCNB2), cyclin dependent kinases (CDK2), cell cycle associated transcription factors (E2F6, E2F7, E2F8) and phosphatases (CDC20, CDC25A, CDC25C) in HCC, which agrees with the existing literature reporting the overexpression of cell cycle related genes in different cancer types including HCC [54–56]. DNA replication during S-phase of cell cycle is one of the crucial step in the completion of cell cycle which also needs supplementation of purines and pyrimidines to build the macromolecules [57]. Similarly, availability of excessive amino acid pool due to decreased catabolic degradation go hand in hand to fulfil the production of protein products in cells undergoing uncontrolled cell division [58]. These existing literature are also in line with our findings that catabolic enzymes involved in amino acid metabolism and, purine and pyrimidine metabolism are downregulated whereas anabolic enzymes are upregulated (Additional file 3: Fig. S2) in HCC as compared to adjacent normal tissues.

Although independent studies have implicated the inhibited metabolic processes and the aberrant cell cycle progression in HCC [59–61], we have shown that both events happen side by side and collectively contribute to the onset and progression of the disease. But we still need to identify the causal relationship between these events; whether suppressed metabolic signaling triggers cell proliferation to efficiently fulfil metabolic tasks resulting in onset and progression of HCC, or it is hyperactive cell cycle signaling which keeps cell’s focus on proliferation rather than performing metabolic processes resulting in onset and progression of HCC.

## Conclusions

Overall, we suggest that simultaneous (1) reversal of hyper-methylation and/or oncogenic miRNA driven suppression of genes involved in metabolic pathways, and (2) induction of hyper-methylation and/or tumor suppressor miRNA driven suppression of genes involved in cell cycle signaling have potential of inhibiting disease aggressiveness, and predicting good survival in HCC.

## Additional files

**Additional file 1: Fig. S1.** Flowchart of constructing meth-miRNA-mRNA network.

**Additional file 2: Table S1.** List of potential tumor suppressors and oncogenes. Left; Tumor suppressors which are downregulated in HCC and whose higher expressions are associated with good survival of HCC patients from TCGA database are listed along with hazard ratio (HR), associated p-value and number of patients (N). Right; Oncogenes which are upregulated in HCC and whose higher expression are associated with poor survival of HCC patients from GEO dataset GSE31384 are listed along with hazard ratio (HR), associated p-value and number of patients (N).

**Additional file 3: Fig. S2.** Metabolic balance between HCC and adjacent normal tissues. Bar graph showing cumulative changes in the expression Z-score values of catabolic (n = 70) and anabolic enzymes (n = 39) between HCC tumors and adjacent normal tissues from meta-analysis of data retrieved from NCBI GEO database (GSE76297, GSE76427, GSE84402, and GSE84598).

**Additional file 4: Table S2.** List of potential tumor suppressor and oncogenic miRNAs. Left; Tumor suppressor miRNAs which are downregulated in HCC and whose higher expressions are associated with good survival of HCC patients from GEO dataset GSE31384 are listed along with hazard ratio (HR), associated p-value and number of patients (N). Right; Oncogenic miRNAs which are upregulated in HCC and whose higher expression are associated with poor survival of HCC patients from GEO dataset GSE31384 are listed along with hazard ratio (HR), associated p-value and number of patients (N).

**Additional file 5: Table S3.** List of targets of tumor suppressor and oncogenic miRNAs in meth-miRNA–mRNA network.

**Additional file 6: Table S4.** List of hypermethylated tumor suppressors and hypomethylated oncogenes in meth-miRNA–mRNA network.

**Additional file 7: Fig. S3.** Hypermethylation driven inhibition of metabolic pathways and hypomethylation driven activation of cell cycle signaling are associated with aggressive disease state in HCC. Dot-plot showing meth-GS scores of HCC patients who experienced or did not experience vascular invasion in TCGA database (**a**). Dot-plot showing meth-GS scores of HCC patients representing different tumor stages (stage I, II or III) in TCGA database (**b**). Dot-plot showing meth-GS scores of HCC patients representing different tumor grades (G1, G2 or G3) in TCGA database (**c**). Dot-plot showing meth-GS scores of HCC patients representing different pathological tumor size (T1, T2 or T3) in TCGA database (**d**). *P < 0.05; **P < 0.01; ns, not significant, Student’s t-test.

**Additional file 8: Fig. S4.** mRNA-GS is associated with less aggressive state in HCC. Dot-plots showing mRNA-GS scores of HCC patients who experienced or did not experience vascular invasion in GEO dataset GSE20017 (**a**) and TCGA database (**b**). Dot-plot showing mRNA-GS scores of HCC patients representing different tumor stages (stage I, II or III) in TCGA database (**c**). Dot-plot showing mRNA-GS scores of HCC patients representing different tumor grades (G1, G2 or G3) in TCGA database (**d**). Dot-plot showing mRNA-GS scores of HCC patients representing different pathological tumor size (T1, T2 or T3) in TCGA database (**e**). *P < 0.05; **P < 0.01; ns, not significant, Student’s t-test.

## Abbreviations

HCC: hepatocellular carcinoma
miRNA: microRNA
meth-miRNA–mRNA: methylation-miRNA–mRNA
NCBI: National Center for Biotechnology Information
GEO: Gene Expression Omnibus
TCGA: The Cancer Genome Atlas
GSEA: Gene Set Enrichment Analysis
TS: tumor suppressor
OG: oncogene
GS: gene signatures
CDK: cyclin-dependent kinase
