# Supplementary figures and images for: Systems biology based meth-miRNA–mRNA regulatory network identifies metabolic imbalance and hyperactive cell cycle signaling involved in hepatocellular carcinoma onset and progression
Source: Cancer Cell Int. 2019 Apr 8;19:89. doi: 10.1186/s12935-019-0804-3 (PMC6454777; doi:10.1186/s12935-019-0804-3)

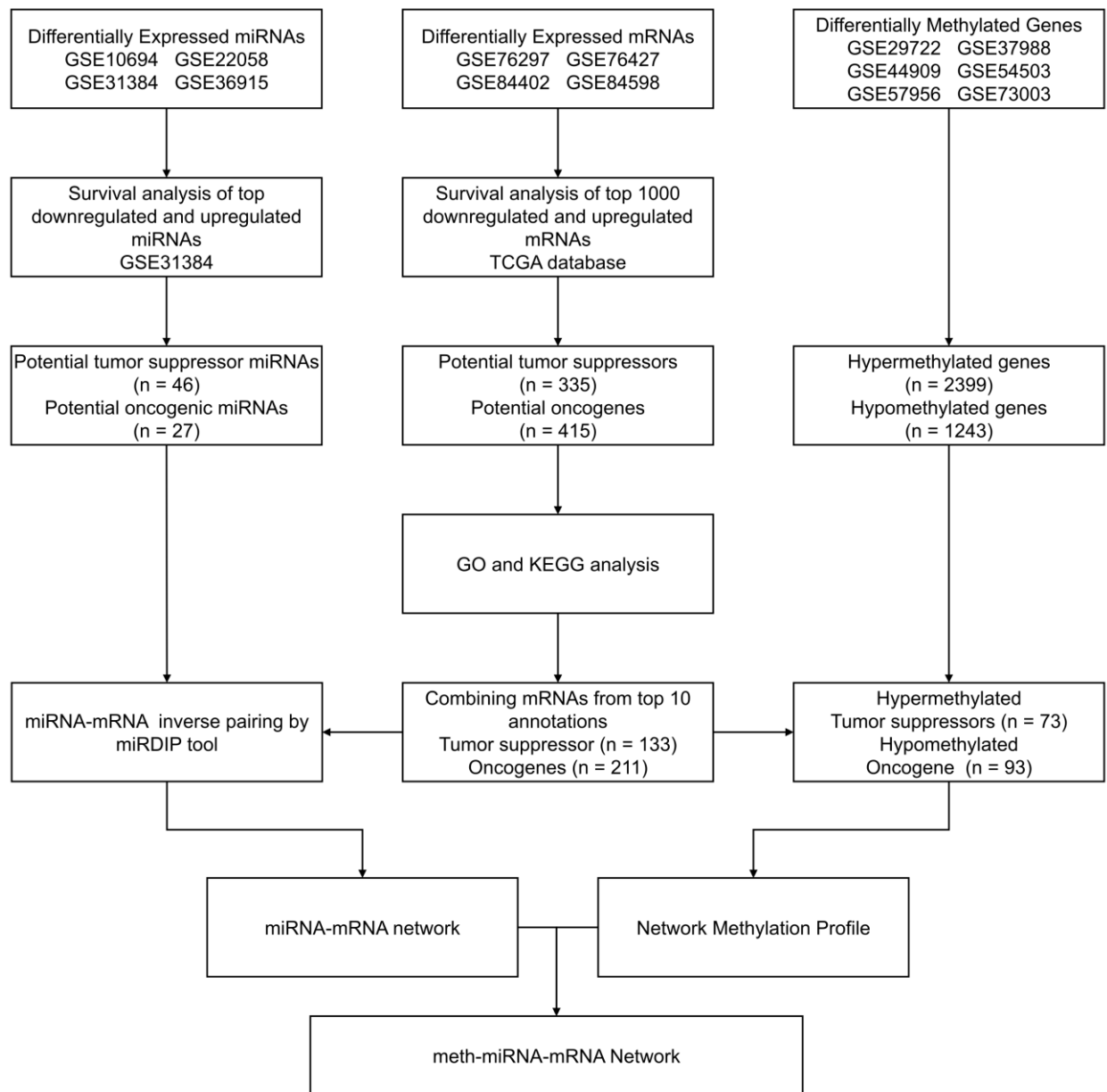

**Fig. S1 Flowchart of constructing meth-miRNA-mRNA network.**

Supplement: Supplementary file 1 — Additional file 1: Fig. S1. Flowchart of constructing meth-miRNA-mRNA network. [file 12935_2019_804_MOESM1_ESM.pdf]
