# Supplementary material for: Systems biology based meth-miRNA–mRNA regulatory network identifies metabolic imbalance and hyperactive cell cycle signaling involved in hepatocellular carcinoma onset and progression
Source: Cancer Cell Int. 2019 Apr 8;19:89. doi: 10.1186/s12935-019-0804-3 (PMC6454777; doi:10.1186/s12935-019-0804-3)
Supplement: Supplementary file 2 — Additional file 2: Table S1. List of potential tumor suppressors and oncogenes. Left; Tumor suppressors which are downregulated in HCC and whose higher expressions are associated with good survival of HCC patients from TCGA database are listed along with hazard ratio (HR), associated p-value and number of patients (N). Right; Oncogenes which are upregulated in HCC and whose higher expression are associated with poor survival of HCC patients from GEO dataset GSE31384 are listed along with hazard ratio (HR), associated p-value and number of patients (N). [file 12935_2019_804_MOESM2_ESM.pdf]

Table S1 List of potential tumor suppressors and oncogenes. Left; Tumor suppressors which are downregulated in HCC and whose higher expressions are associated with good survival of HCC patients from TCGA database are listed along with hazard ratio (HR), associated p-value and number of patients (N). Right; Oncogenes which are upregulated in HCC and whose higher expression are associated with poor survival of HCC patients from GEO dataset GSE31384 are listed along with hazard ratio (HR), associated p-value and number of patients (N).

Tumor Suppressor	HR	p-value	N	Oncogene	HR	p-value	N
**RAMP3**	0.43	2.63E-06	360	**CDC20**	2.62	1.41E-07	360
**DNASE1L3**	0.44	4.86E-06	360	**HN1**	2.52	3.8E-07	360
**SOCS2**	0.44	6.25E-06	360	**CDCA8**	2.47	5.63E-07	360
**CRY2**	0.45	1.03E-05	360	**GTPBP4**	2.40	1.34E-06	360
**DMGDH**	0.45	0.000012	360	**NEIL3**	2.32	3.14E-06	360
**PPARGC1A**	0.45	0.000015	360	**KIF2C**	2.26	5.81E-06	360
**SLC2A2**	0.47	2.29E-05	360	**BIRC5**	2.26	7.43E-06	360
**CFHR3**	0.47	3.25E-05	360	**TRIP13**	2.24	7.99E-06	360
**HMGCS2**	0.47	3.33E-05	360	**SLC41A3**	2.23	8.5E-06	360
**PSD4**	0.48	3.61E-05	360	**CSTF2**	2.21	9.28E-06	360
**NDRG2**	0.48	0.000040	360	**SPATS2**	2.18	1.55E-05	360
**PON1**	0.48	4.17E-05	360	**TMEM106C**	2.17	1.66E-05	360
**FYN**	0.48	4.28E-05	360	**CENPA**	2.16	2.05E-05	360
**ITIH1**	0.48	5.34E-05	360	**EZH2**	2.16	2.26E-05	360
**SLC22A1**	0.48	5.95E-05	360	**SPC25**	2.14	2.47E-05	360
**ROBO4**	0.49	6.02E-05	360	**DR1**	2.14	3.04E-05	360
**CPEB3**	0.49	0.000063	360	**RAD54L**	2.12	2.89E-05	360
**GOT2**	0.49	6.33E-05	360	**CENPH**	2.12	3.11E-05	360
**DHRS1**	0.50	0.000106	360	**WDR53**	2.11	3.48E-05	360
**MGMT**	0.50	0.000135	360	**E2F8**	2.10	3.66E-05	360
**ADRA2B**	0.51	0.000126	360	**CENPL**	2.10	0.000037	360
**GNA14**	0.51	0.000146	360	**HMMR**	2.09	3.91E-05	360
**GIMAP7**	0.51	0.000158	360	**LIN9**	2.08	5.35E-05	360
**IQGAP2**	0.51	0.000160	360	**SF3B4**	2.07	5.01E-05	360
**TPPP2**	0.51	0.000163	360	**SMG5**	2.07	0.000051	360
**MYCT1**	0.51	0.000163	360	**MPV17**	2.05	6.27E-05	360
**UPB1**	0.51	0.000166	360	**TIPIN**	2.05	7.21E-05	360
**MASP1**	0.52	0.000222	360	**RAB10**	2.04	6.39E-05	360
**TMEM220**	0.52	0.000239	360	**SGOL2**	2.04	6.94E-05	360
**TAT**	0.52	0.000254	360	**CDC7**	2.04	7.03E-05	360
**ADH4**	0.52	0.000259	360	**KIAA0101**	2.04	0.000076	360
**ENG**	0.52	0.000269	360	**EEF1E1**	2.04	9.68E-05	360
**LIMS2**	0.52	0.000290	360	**KIFC1**	2.02	8.92E-05	360
**APOB**	0.52	0.000296	360	**PA2G4**	2.02	0.000097	360
**SLC10A1**	0.52	0.000298	360	**SKA1**	2.01	0.000120	360
**IL18RAP**	0.52	0.000313	360	**SGOL1**	2.00	0.000118	360
**GP6**	0.52	0.000334	360	**LAPTM4B**	2.00	0.000159	360
**CCDC69**	0.53	0.000454	360	**KIF15**	1.99	0.000111	360
**ASPA**	0.53	0.000475	360	**MRPS23**	1.99	0.000126	360
**CYP3A43**	0.54	0.000542	360	**CCNB1**	1.99	0.000141	360
**FTCD**	0.54	0.000550	360	**MAD2L1**	1.98	0.000141	360
**CYP2C19**	0.54	0.000557	360	**E2F6**	1.98	0.000146	360
**AFM**	0.54	0.000557	360	**MAPKAPK5**	1.98	0.000148	360
**ESR1**	0.54	0.000561	360	**NAT10**	1.97	0.000143	360
**GYS2**	0.54	0.000574	360	**CPSF3**	1.97	0.000156	360
**CYP2C9**	0.54	0.000583	360	**E2F7**	1.97	0.000157	360
**GABARAPL1**	0.54	0.000597	360	**CCNF**	1.97	0.000161	360

Tumor Suppressor	HR	p-value	N
**APOC1**	0.54	0.000660	360
**CSAD**	0.54	0.000666	360
**CAT**	0.54	0.000681	360
**CYB5D2**	0.54	0.000684	360
**SARDH**	0.54	0.000710	360
**CPN2**	0.54	0.000720	360
**ABCA9**	0.54	0.000779	360
**PTPRB**	0.55	0.000741	360
**IGFALS**	0.55	0.000757	360
**GIMAP8**	0.55	0.000765	360
**CYP2C8**	0.55	0.000801	360
**IVD**	0.55	0.000829	360
**MUT**	0.55	0.000860	360
**SPP2**	0.55	0.000881	360
**TMPRSS6**	0.55	0.000945	360
**DECR1**	0.55	0.000989	360
**TCF21**	0.56	0.000986	360
**N4BP2L1**	0.56	0.001045	360
**PIK3R1**	0.56	0.001174	360
**CCL23**	0.56	0.001283	360
**SLC31A1**	0.56	0.001285	360
**EOMES**	0.56	0.001287	360
**ADRA1A**	0.56	0.001299	360
**STARD5**	0.56	0.001325	360
**ETS2**	0.56	0.001342	360
**MMAA**	0.56	0.001455	360
**ETFDH**	0.56	0.001458	360
**CYP3A5**	0.57	0.001433	360
**GPT**	0.57	0.001489	360
**ANKRD24**	0.57	0.001554	360
**NTF3**	0.57	0.001772	360
**F9**	0.57	0.001809	360
**CCT6B**	0.57	0.001925	360
**LDB2**	0.58	0.001949	360
**FGB**	0.58	0.002011	360
**SLC41A2**	0.58	0.002098	360
**GPR182**	0.58	0.002109	360
**SLCO2B1**	0.58	0.002148	360
**C8B**	0.58	0.002151	360
**ZAP70**	0.58	0.002189	360
**HPX**	0.58	0.002236	360
**CYP4F12**	0.58	0.002268	360
**C2orf40**	0.58	0.002348	360
**ANGPTL1**	0.58	0.002443	360
**NKG7**	0.58	0.002453	360
**GHR**	0.58	0.002509	360
**FAM65C**	0.58	0.002527	360
**TAPT1**	0.58	0.002580	360
**FAHD2A**	0.58	0.002589	360
**SPRYD4**	0.58	0.002671	360
**SERPINA4**	0.58	0.002739	360
**SLC25A11**	0.58	0.002854	360
**ACOT12**	0.59	0.002709	360

Oncogene	HR	p-value	N
**STMN1**	1.97	0.000188	360
**MCM6**	1.96	0.000181	360
**NUF2**	1.95	0.000193	360
**ATIC**	1.94	0.000215	360
**TMCO3**	1.93	0.000245	360
**DLGAP5**	1.93	0.000264	360
**PIGU**	1.93	0.000280	360
**HJURP**	1.92	0.000264	360
**EFNA4**	1.92	0.000276	360
**NEDD1**	1.92	0.000293	360
**PPM1G**	1.92	0.000366	360
**CAD**	1.91	0.000284	360
**POLQ**	1.91	0.000293	360
**MCM7**	1.91	0.000308	360
**VPS26A**	1.91	0.000336	360
**CKAP5**	1.90	0.000323	360
**CSE1L**	1.90	0.000343	360
**TTK**	1.89	0.000372	360
**OIP5**	1.89	0.000400	360
**PSMD1**	1.89	0.000459	360
**RABIF**	1.88	0.000445	360
**PLK1**	1.88	0.000504	360
**PTGES3**	1.88	0.000508	360
**KIF20A**	1.87	0.000460	360
**FSD1L**	1.87	0.000479	360
**KIF11**	1.87	0.000500	360
**SESTD1**	1.87	0.000503	360
**AGFG1**	1.87	0.000534	360
**ANLN**	1.86	0.000505	360
**MCM2**	1.86	0.000512	360
**EXO1**	1.86	0.000532	360
**HDAC2**	1.86	0.000586	360
**SRD5A3**	1.86	0.000652	360
**TRAIP**	1.85	0.000596	360
**RRP15**	1.85	0.000671	360
**ISY1**	1.85	0.000715	360
**SNRPD1**	1.84	0.000654	360
**RBM45**	1.84	0.000697	360
**RAD51AP1**	1.83	0.000722	360
**TRMT6**	1.83	0.000735	360
**VPS72**	1.83	0.000790	360
**FARSB**	1.82	0.000781	360
**MELK**	1.82	0.000794	360
**CHEK1**	1.82	0.000809	360
**RAE1**	1.82	0.000817	360
**VRK2**	1.82	0.000820	360
**PRR11**	1.82	0.000829	360
**MSH2**	1.81	0.000928	360
**CCNA2**	1.81	0.000991	360
**SNRPB2**	1.81	0.000999	360
**H2AFZ**	1.81	0.001018	360
**DPH2**	1.80	0.000931	360
**DEPDC1**	1.80	0.000950	360

Tumor Suppressor	HR	p-value	N
**DHX58**	0.59	0.002785	360
**SLC38A4**	0.59	0.002843	360
**IL33**	0.59	0.002916	360
**GFRA2**	0.59	0.002919	360
**IGF1**	0.59	0.002940	360
**RDH16**	0.59	0.002947	360
**FBLN5**	0.59	0.003139	360
**AKR1D1**	0.59	0.003242	360
**RNF125**	0.59	0.003282	360
**DBT**	0.59	0.003348	360
**FCGRT**	0.59	0.003373	360
**AVPR2**	0.59	0.003402	360
**SLCO1B1**	0.59	0.003508	360
**GLYATL1**	0.59	0.003861	360
**COLEC10**	0.60	0.003631	360
**GZMH**	0.60	0.003701	360
**FMO3**	0.60	0.003869	360
**STEAP4**	0.60	0.003998	360
**PON3**	0.60	0.004098	360
**APOC3**	0.60	0.004123	360
**HAGH**	0.60	0.004127	360
**MPDZ**	0.60	0.004186	360
**FAM149A**	0.60	0.004186	360
**SHPK**	0.60	0.004294	360
**DPYS**	0.60	0.004474	360
**ENPEP**	0.60	0.004475	360
**RHOB**	0.60	0.004487	360
**MYOM2**	0.60	0.004527	360
**DEPDC7**	0.60	0.004532	360
**KLF2**	0.60	0.004540	360
**AMDHD1**	0.60	0.004574	360
**PBLD**	0.60	0.004876	360
**KLRB1**	0.60	0.004914	360
**SLC17A2**	0.60	0.004985	360
**ADH1A**	0.60	0.005012	360
**GCDH**	0.60	0.005016	360
**ADHFE1**	0.60	0.005255	360
**GNMT**	0.61	0.004836	360
**APOA1**	0.61	0.005015	360
**HPN**	0.61	0.005058	360
**GADD45A**	0.61	0.005166	360
**ACAT1**	0.61	0.005449	360
**SELP**	0.61	0.005557	360
**CST7**	0.61	0.005648	360
**ACSM3**	0.61	0.005693	360
**C16orf45**	0.61	0.005962	360
**PDE7B**	0.61	0.005966	360
**GADD45G**	0.61	0.006086	360
**LECT2**	0.61	0.006138	360
**METTL7A**	0.61	0.006147	360
**FDX1**	0.61	0.006232	360
**ADAMTSL2**	0.61	0.006236	360
**AVPI1**	0.61	0.006248	360

Oncogene	HR	p-value	N
**DUSP12**	1.80	0.001004	360
**ITGB1BP1**	1.80	0.001097	360
**TMEM206**	1.80	0.001197	360
**KIAA1524**	1.79	0.001080	360
**APEX2**	1.79	0.001109	360
**AATF**	1.79	0.001130	360
**PRC1**	1.79	0.001133	360
**UBE2T**	1.79	0.001206	360
**BTF3L4**	1.79	0.001213	360
**EED**	1.78	0.001206	360
**GMPS**	1.78	0.001254	360
**HSF2BP**	1.78	0.001260	360
**PBK**	1.78	0.001268	360
**ZNF792**	1.78	0.001287	360
**NUP85**	1.78	0.001303	360
**SLC39A1**	1.78	0.001319	360
**HDAC1**	1.78	0.001365	360
**NDC80**	1.77	0.001330	360
**CCT4**	1.77	0.001477	360
**CKAP2L**	1.77	0.001484	360
**CEP55**	1.77	0.001490	360
**STIP1**	1.76	0.001582	360
**ARF4**	1.76	0.001587	360
**STIL**	1.76	0.001595	360
**ATP6V1E1**	1.76	0.001614	360
**SLC39A10**	1.76	0.001774	360
**PRIM1**	1.75	0.001683	360
**NUP205**	1.75	0.001683	360
**SNRPE**	1.75	0.001685	360
**GINS1**	1.75	0.001749	360
**CCDC34**	1.75	0.001915	360
**TOP2A**	1.74	0.001849	360
**CENPK**	1.74	0.001865	360
**CLIC1**	1.74	0.001939	360
**RRM2**	1.74	0.001960	360
**KIF14**	1.73	0.002139	360
**GTF3C2**	1.73	0.002160	360
**NME6**	1.73	0.002175	360
**PAK1IP1**	1.73	0.002234	360
**PIGC**	1.73	0.002286	360
**PTTG1**	1.73	0.002290	360
**MKI67**	1.73	0.002326	360
**SLC39A6**	1.72	0.002374	360
**CENPI**	1.72	0.002381	360
**ILF2**	1.72	0.002418	360
**NCAPG**	1.72	0.002454	360
**FAM83D**	1.72	0.002473	360
**EIF2S2**	1.72	0.002491	360
**XPOT**	1.71	0.002564	360
**ZNF605**	1.71	0.002575	360
**FLVCR1**	1.71	0.002605	360
**EME1**	1.71	0.002667	360
**ECT2**	1.71	0.002705	360

Tumor Suppressor	HR	p-value	N
**QDPR**	0.61	0.006649	360
**ACADS**	0.61	0.006703	360
**MAN2B2**	0.61	0.006909	360
**GFOD1**	0.62	0.006531	360
**LMO2**	0.62	0.006596	360
**XDH**	0.62	0.006735	360
**TK2**	0.62	0.006991	360
**NR1I2**	0.62	0.007181	360
**OIT3**	0.62	0.007213	360
**GPR146**	0.62	0.007250	360
**KBTBD11**	0.62	0.007269	360
**GPT2**	0.62	0.007317	360
**C5**	0.62	0.007400	360
**RANBP3L**	0.62	0.007422	360
**ADI1**	0.62	0.007508	360
**CYP27A1**	0.62	0.007588	360
**HGD**	0.62	0.007617	360
**F7**	0.62	0.007711	360
**PRICKLE1**	0.62	0.007797	360
**PROZ**	0.62	0.007879	360
**F2**	0.62	0.007900	360
**GRAMD1C**	0.62	0.007910	360
**CRHBP**	0.62	0.007958	360
**C1RL**	0.62	0.007992	360
**RBP4**	0.62	0.008011	360
**TTC36**	0.62	0.008107	360
**PGM1**	0.62	0.008218	360
**SLC46A3**	0.62	0.008227	360
**GAMT**	0.62	0.008308	360
**FARP2**	0.62	0.008367	360
**CYP8B1**	0.62	0.008456	360
**DENND1C**	0.62	0.008842	360
**ACBD4**	0.63	0.008420	360
**CD226**	0.63	0.008949	360
**CIDEB**	0.63	0.008979	360
**COBLL1**	0.63	0.009022	360
**SDS**	0.63	0.009033	360
**C1orf168**	0.63	0.009170	360
**PLAC9**	0.63	0.009386	360
**MSRA**	0.63	0.009441	360
**SLC16A4**	0.63	0.009441	360
**UROC1**	0.63	0.009571	360
**C7**	0.63	0.009641	360
**ADH1C**	0.63	0.009685	360
**TNFRSF1B**	0.63	0.009696	360
**ANO1**	0.63	0.009768	360
**GPM6A**	0.63	0.010304	360
**PIPOX**	0.63	0.010580	360
**CLDN14**	0.63	0.010616	360
**DHTKD1**	0.63	0.011292	360
**SLC25A42**	0.63	0.011726	360
**CBFA2T3**	0.64	0.011022	360
**GLYCTK**	0.64	0.011116	360

Oncogene	HR	p-value	N
**CDCA2**	1.71	0.002706	360
**SCML2**	1.71	0.002711	360
**CPSF6**	1.71	0.002801	360
**YWHAQ**	1.71	0.002905	360
**NARS**	1.71	0.002933	360
**RFC4**	1.70	0.002938	360
**LCLAT1**	1.70	0.002954	360
**CDC6**	1.70	0.002957	360
**BRIX1**	1.70	0.003173	360
**DAP3**	1.70	0.003179	360
**YEATS2**	1.70	0.003355	360
**SEC61G**	1.69	0.003298	360
**CEP250**	1.69	0.003317	360
**MASTL**	1.69	0.003318	360
**CHAF1B**	1.69	0.003339	360
**CDC123**	1.69	0.003387	360
**GRPEL2**	1.69	0.003444	360
**AURKB**	1.69	0.003499	360
**PDIA6**	1.69	0.003532	360
**PSMD14**	1.68	0.003331	360
**RAB23**	1.68	0.003648	360
**CCT7**	1.68	0.003664	360
**C17orf80**	1.68	0.003717	360
**CDC25A**	1.68	0.003794	360
**LMNB1**	1.68	0.003822	360
**CASP2**	1.67	0.003937	360
**PDCD10**	1.67	0.003958	360
**PPIH**	1.67	0.003994	360
**ANXA2**	1.67	0.004087	360
**HMGB2**	1.67	0.004369	360
**PPAT**	1.66	0.004628	360
**SART3**	1.66	0.004629	360
**RNASEH2A**	1.66	0.004662	360
**RIT1**	1.66	0.004716	360
**KIF2A**	1.66	0.004785	360
**NUP155**	1.66	0.004829	360
**SEH1L**	1.66	0.005107	360
**CDC73**	1.66	0.005865	360
**SUPT16H**	1.65	0.004809	360
**FEN1**	1.65	0.004935	360
**CENPE**	1.65	0.004978	360
**CDC25C**	1.65	0.005063	360
**NCL**	1.65	0.005082	360
**EFTUD2**	1.65	0.005143	360
**DARS2**	1.65	0.005267	360
**S100A10**	1.65	0.005360	360
**TBL1XR1**	1.65	0.005755	360
**UTP6**	1.64	0.005210	360
**NUP43**	1.64	0.005316	360
**CDCA4**	1.64	0.005495	360
**GLMN**	1.64	0.005587	360
**RUVBL1**	1.64	0.005610	360
**POLR3C**	1.64	0.005627	360

Tumor Suppressor	HR	p-value	N
**ABHD14B**	0.64	0.011434	360
**ALDH2**	0.64	0.011643	360
**SPSB3**	0.64	0.011647	360
**HGFAC**	0.64	0.011770	360
**CHST7**	0.64	0.012011	360
**CBR4**	0.64	0.012632	360
**EPM2A**	0.64	0.012670	360
**NPY1R**	0.64	0.012757	360
**RCAN1**	0.64	0.012830	360
**ASGR2**	0.64	0.013039	360
**CYP4V2**	0.64	0.013047	360
**C6**	0.64	0.013393	360
**ACSM5**	0.64	0.013402	360
**CHRNE**	0.64	0.014125	360
**POU6F1**	0.64	0.014131	360
**HDAC6**	0.64	0.014730	360
**SLC9A3R2**	0.65	0.013994	360
**CYP4F2**	0.65	0.014041	360
**GGT5**	0.65	0.014402	360
**LYRM5**	0.65	0.014511	360
**HAO1**	0.65	0.014643	360
**ECHS1**	0.65	0.015438	360
**KIAA0922**	0.65	0.015584	360
**HEBP1**	0.65	0.015820	360
**DLG2**	0.65	0.016034	360
**RIPK4**	0.65	0.016353	360
**DGAT2**	0.65	0.017572	360
**ZCCHC24**	0.65	0.017861	360
**ALDH8A1**	0.65	0.018499	360
**BMPER**	0.65	0.018607	360
**SERPINA10**	0.66	0.017776	360
**KLKB1**	0.66	0.017786	360
**OGDHL**	0.66	0.017842	360
**LDHD**	0.66	0.018291	360
**ACSM2A**	0.66	0.018702	360
**NCAN**	0.66	0.019018	360
**EPHX2**	0.66	0.019345	360
**MRPL54**	0.66	0.019496	360
**SPAG7**	0.66	0.019670	360
**HAO2**	0.66	0.020178	360
**PZP**	0.66	0.020356	360
**ABCA8**	0.66	0.020442	360
**ALDH9A1**	0.66	0.020838	360
**CBS**	0.66	0.020855	360
**SLC27A2**	0.66	0.020891	360
**ALPL**	0.66	0.021447	360
**ZNF18**	0.66	0.021735	360
**CFP**	0.66	0.022201	360
**CYP2E1**	0.66	0.022258	360
**SEC14L2**	0.66	0.022323	360
**NDST3**	0.66	0.022481	360
**FBP1**	0.67	0.022517	360
**SAT2**	0.67	0.022959	360

Oncogene	HR	p-value	N
**COMMD2**	1.64	0.005655	360
**TAF6**	1.64	0.005796	360
**RIOK1**	1.64	0.006393	360
**PRMT3**	1.63	0.005938	360
**C5orf34**	1.63	0.005950	360
**TTLL4**	1.63	0.006107	360
**BTBD10**	1.63	0.006212	360
**NDRG3**	1.63	0.006218	360
**CCT2**	1.63	0.006235	360
**DTL**	1.63	0.006310	360
**LRRC42**	1.63	0.006426	360
**GOLT1B**	1.63	0.006428	360
**HNRNPR**	1.63	0.006698	360
**NCAPG2**	1.63	0.006776	360
**GPATCH4**	1.63	0.006827	360
**CENPQ**	1.63	0.006852	360
**IARS**	1.62	0.006357	360
**HSF2**	1.62	0.006562	360
**MYO19**	1.62	0.006767	360
**SNRPA**	1.62	0.006956	360
**APAF1**	1.62	0.007059	360
**CBFA2T2**	1.62	0.007062	360
**CDCA3**	1.62	0.007063	360
**PHOSPHO2**	1.62	0.007526	360
**DYNC1LI1**	1.61	0.007658	360
**POLA2**	1.61	0.007696	360
**YIF1B**	1.61	0.007699	360
**PSPH**	1.61	0.007888	360
**DRG1**	1.61	0.007989	360
**C5orf28**	1.61	0.008004	360
**KCMF1**	1.61	0.008181	360
**HSPA14**	1.61	0.008197	360
**XRN2**	1.61	0.008724	360
**HMGN4**	1.60	0.008376	360
**BRD8**	1.60	0.008417	360
**C17orf53**	1.60	0.008698	360
**PPIA**	1.60	0.008704	360
**XPR1**	1.60	0.008720	360
**DCTN2**	1.60	0.008758	360
**NKIRAS2**	1.60	0.008762	360
**TXNDC9**	1.60	0.008795	360
**STAM**	1.60	0.008799	360
**CBX1**	1.60	0.009036	360
**EIF4A3**	1.60	0.009200	360
**KIAA0100**	1.59	0.008885	360
**TDG**	1.59	0.008978	360
**FANCI**	1.59	0.008983	360
**TK1**	1.59	0.009343	360
**KPNB1**	1.59	0.009446	360
**NVL**	1.58	0.010095	360
**AURKA**	1.58	0.010270	360
**UBE2C**	1.58	0.011131	360
**AGBL5**	1.57	0.010769	360

Tumor Suppressor	HR	p-value	N
**DAO**	0.67	0.023178	360
**WFDC1**	0.67	0.023474	360
**ERRFI1**	0.67	0.023666	360
**CD247**	0.67	0.023949	360
**HAPLN2**	0.67	0.023962	360
**GLOD4**	0.67	0.024175	360
**AGXT**	0.67	0.024443	360
**IGFBP4**	0.67	0.024604	360
**CNGA1**	0.67	0.024803	360
**HAAO**	0.67	0.025158	360
**PCOLCE**	0.67	0.025488	360
**SUCLG2**	0.67	0.025530	360
**SLC27A5**	0.67	0.025615	360
**GBA3**	0.67	0.025959	360
**DBH**	0.67	0.026083	360
**C8A**	0.67	0.026126	360
**DNMT3L**	0.67	0.026161	360
**NOSTRIN**	0.67	0.026431	360
**ZCCHC6**	0.67	0.027158	360
**PHYHD1**	0.67	0.027454	360
**DLC1**	0.67	0.027638	360
**NR3C2**	0.68	0.027902	360
**SRD5A2**	0.68	0.028050	360
**CXCL2**	0.68	0.028134	360
**PCK2**	0.68	0.028747	360
**LAMB2**	0.68	0.029266	360
**AGXT2**	0.68	0.029509	360
**AKR7A3**	0.68	0.029517	360
**CD14**	0.68	0.029733	360
**ABCA6**	0.68	0.030049	360
**ADRA1B**	0.68	0.030209	360
**LTK**	0.68	0.030644	360
**MAMDC4**	0.68	0.031178	360
**ICAM3**	0.68	0.031445	360
**NTHL1**	0.68	0.031607	360
**CYP4A11**	0.68	0.031680	360
**HADH**	0.68	0.031734	360
**LEAP2**	0.68	0.032371	360
**SDPR**	0.68	0.033183	360
**FMO2**	0.68	0.033340	360
**LIME1**	0.68	0.033797	360
**MT1X**	0.68	0.034117	360
**TMBIM6**	0.68	0.034229	360
**F12**	0.69	0.033348	360
**PEX11G**	0.69	0.033937	360
**MAN1C1**	0.69	0.034668	360
**CYP4F3**	0.69	0.037175	360
**HABP2**	0.69	0.037757	360
**MACROD1**	0.69	0.037923	360
**MOGAT1**	0.69	0.038079	360
**MEIS3P1**	0.69	0.038182	360
**DCN**	0.69	0.038297	360
**CNDP1**	0.69	0.038355	360

Oncogene	HR	p-value	N
**TMEM185B**	1.57	0.010892	360
**AHSA1**	1.57	0.010909	360
**EIF2B1**	1.57	0.010948	360
**CAPRIN1**	1.57	0.011326	360
**USP39**	1.57	0.011433	360
**FAM127A**	1.57	0.011633	360
**MCM3**	1.57	0.011790	360
**PPIL1**	1.57	0.011832	360
**HPS3**	1.57	0.011865	360
**DCK**	1.57	0.011943	360
**C1orf112**	1.57	0.012041	360
**CENPJ**	1.57	0.012256	360
**TIPRL**	1.56	0.012443	360
**ENAH**	1.56	0.012609	360
**THOC7**	1.56	0.012750	360
**PSMA1**	1.56	0.012911	360
**FUBP1**	1.56	0.012954	360
**ME2**	1.56	0.013071	360
**MND1**	1.56	0.013186	360
**HNRNPM**	1.56	0.013966	360
**MRPL9**	1.55	0.013449	360
**KIF20B**	1.55	0.013582	360
**WDR70**	1.55	0.013644	360
**DCLRE1B**	1.55	0.013685	360
**GINS4**	1.55	0.014129	360
**TAF1B**	1.55	0.014140	360
**ZNF346**	1.55	0.014159	360
**DENND5A**	1.55	0.014233	360
**TLCD1**	1.55	0.014264	360
**NUP37**	1.55	0.014333	360
**SSB**	1.55	0.014599	360
**ASF1B**	1.55	0.014772	360
**PUS7**	1.55	0.014790	360
**CDV3**	1.55	0.014868	360
**EDC3**	1.54	0.014938	360
**NME7**	1.54	0.015014	360
**SENP1**	1.54	0.015112	360
**DCAF16**	1.54	0.015162	360
**TMEM147**	1.54	0.015659	360
**TULP3**	1.54	0.015857	360
**DDX1**	1.54	0.015888	360
**UTP14A**	1.54	0.015923	360
**WDR12**	1.54	0.016064	360
**TSEN15**	1.54	0.016187	360
**GMFB**	1.54	0.016854	360
**HTRA2**	1.54	0.017197	360
**CKS2**	1.53	0.016398	360
**RFWD3**	1.53	0.016498	360
**ACTR5**	1.53	0.016509	360
**CCT6A**	1.53	0.016572	360
**NAT9**	1.53	0.016783	360
**TPRKB**	1.53	0.016788	360
**ZNF84**	1.53	0.016812	360

Tumor Suppressor	HR	p-value	N
**ABHD6**	0.69	0.038451	360
**JDP2**	0.69	0.038514	360
**ORM1**	0.69	0.038851	360
**ALAS1**	0.69	0.038976	360
**BAZ2B**	0.69	0.039518	360
**FOXF1**	0.69	0.039601	360
**SLC25A34**	0.69	0.039896	360
**NAT2**	0.69	0.040466	360
**MST1**	0.69	0.040503	360
**LHPP**	0.69	0.040578	360
**CA5A**	0.69	0.041395	360
**HELQ**	0.69	0.042232	360
**TTR**	0.69	0.042664	360
**CDC37L1**	0.70	0.042164	360
**FGA**	0.70	0.042482	360
**ADH6**	0.70	0.042544	360
**CFB**	0.70	0.042802	360
**PCK1**	0.70	0.043572	360
**HPD**	0.70	0.043575	360
**INMT**	0.70	0.043603	360
**FCN2**	0.70	0.043875	360
**EFCAB6**	0.70	0.044216	360
**SIRT5**	0.70	0.044363	360

Oncogene	HR	p-value	N
**ATP7A**	1.53	0.016904	360
**LBR**	1.53	0.016974	360
**LYAR**	1.53	0.017027	360
**CCNB2**	1.53	0.017152	360
**TKT**	1.53	0.017253	360
**UTP18**	1.53	0.017262	360
**TIMELESS**	1.53	0.017437	360
**FASTKD3**	1.53	0.017725	360
**POLR3F**	1.53	0.017976	360
**SSR3**	1.53	0.018008	360
**HAT1**	1.52	0.017937	360
**CDC23**	1.52	0.018227	360
**STT3A**	1.52	0.018969	360
**PDSS1**	1.52	0.019231	360
**H2AFY**	1.52	0.019370	360
**PRKAG1**	1.52	0.019405	360
**RHEB**	1.52	0.019661	360
**ENOPH1**	1.52	0.01968	360
**VPS35**	1.51	0.019845	360
**CDK2**	1.51	0.020123	360
**GTPBP2**	1.51	0.020138	360
**BUB3**	1.51	0.020205	360
**CPSF4**	1.51	0.021305	360
**RPIA**	1.51	0.021343	360
**WDYHV1**	1.51	0.021679	360
**B4GALT3**	1.51	0.021691	360
**GARS**	1.51	0.021956	360
**TMEM69**	1.5	0.021586	360
**FANCF**	1.50	0.021597	360
**ANAPC7**	1.50	0.021732	360
**TCHP**	1.50	0.022475	360
**DVL3**	1.50	0.022594	360
**PSMB5**	1.50	0.023292	360
**PCNA**	1.50	0.023368	360
**ASF1A**	1.50	0.024190	360
**CPNE1**	1.50	0.024210	360
**RNASEH1**	1.49	0.024661	360
**DEDD**	1.49	0.024884	360
**NAE1**	1.49	0.025059	360
**DPF2**	1.49	0.025342	360
**UBE2O**	1.49	0.025871	360
**AGPS**	1.49	0.026993	360
**H3F3A**	1.49	0.027916	360
**FIGNL1**	1.48	0.026747	360
**SLC38A6**	1.48	0.026822	360
**PRKDC**	1.48	0.027270	360
**POLE2**	1.48	0.027450	360
**SQLE**	1.48	0.027924	360
**CNOT10**	1.48	0.028151	360
**DNMT1**	1.48	0.028350	360
**SNX5**	1.48	0.028626	360
**C4orf46**	1.48	0.028647	360
**METTL6**	1.48	0.028813	360

Oncogene	HR	p-value	N
**DEK**	1.48	0.029079	360
**TPR**	1.48	0.029757	360
**SF3B1**	1.48	0.030374	360
**WDSUB1**	1.47	0.029298	360
**TOPBP1**	1.47	0.029425	360
**COPS7B**	1.47	0.029550	360
**SLC4A1AP**	1.47	0.030062	360
**EIF2B4**	1.47	0.030141	360
**NOL11**	1.47	0.030726	360
**ITGB3BP**	1.47	0.030981	360
**PTPDC1**	1.47	0.031169	360
**ACTR10**	1.47	0.031329	360
**TMCO1**	1.47	0.031575	360
**NCBP2**	1.47	0.032696	360
**PRKAA2**	1.46	0.031617	360
**MSTO1**	1.46	0.032622	360
**NPC1**	1.46	0.033519	360
**CNIH4**	1.46	0.033581	360
**CALU**	1.46	0.033855	360
**VPS8**	1.46	0.033884	360
**DYNLL1**	1.46	0.034200	360
**NUP107**	1.46	0.034907	360
**MCM5**	1.46	0.035083	360
**ADSL**	1.46	0.035761	360
**ANO10**	1.45	0.035167	360
**ARL8B**	1.45	0.036124	360
**ZFP64**	1.45	0.036236	360
**SDF2**	1.45	0.036270	360
**DNM1L**	1.45	0.036374	360
**RAP2A**	1.45	0.036384	360
**TFRC**	1.45	0.036514	360
**SAP130**	1.45	0.036524	360
**VPS45**	1.45	0.037381	360
**WDR55**	1.45	0.037390	360
**TTC27**	1.45	0.037794	360
**EXOSC2**	1.45	0.038312	360
**LARP4B**	1.45	0.038348	360
**SETDB1**	1.45	0.038623	360
**FAF1**	1.45	0.039699	360
**CDK5**	1.45	0.039762	360
**WNK1**	1.44	0.039692	360
**DLG5**	1.44	0.039994	360
**RBM12B**	1.44	0.040101	360
**EIF2S3**	1.44	0.040317	360
**C2orf44**	1.44	0.040466	360
**CSNK2A1**	1.44	0.042146	360
**UBAP2L**	1.44	0.044350	360
**VRK1**	1.43	0.043041	360
**POLR2H**	1.43	0.043964	360
**CHRAC1**	1.43	0.044215	360
