# Supplementary material for: Systems biology based meth-miRNA–mRNA regulatory network identifies metabolic imbalance and hyperactive cell cycle signaling involved in hepatocellular carcinoma onset and progression
Source: Cancer Cell Int. 2019 Apr 8;19:89. doi: 10.1186/s12935-019-0804-3 (PMC6454777; doi:10.1186/s12935-019-0804-3)
Supplement: Supplementary file 3 — Additional file 3: Fig. S2. Metabolic balance between HCC and adjacent normal tissues. Bar graph showing cumulative changes in the expression Z-score values of catabolic (n = 70) and anabolic enzymes (n = 39) between HCC tumors and adjacent normal tissues from meta-analysis of data retrieved from NCBI GEO database (GSE76297, GSE76427, GSE84402, and GSE84598). [file 12935_2019_804_MOESM3_ESM.pdf]

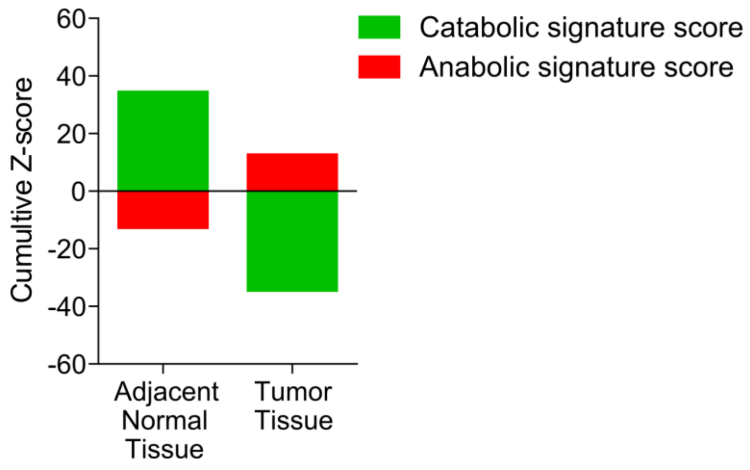

Fig. S2 Metabolic balance between HCC and adjacent normal tissues. Bar graph showing cumulative changes in the expression Z-score values of catabolic (n = 70) and anabolic enzymes (n = 39) between HCC tumors and adjacent normal tissues from meta-analysis of data retrieved from NCBI GEO database (GSE76297, GSE76427, GSE84402, and GSE84598).
