# Supplementary material for: Systems biology based meth-miRNA–mRNA regulatory network identifies metabolic imbalance and hyperactive cell cycle signaling involved in hepatocellular carcinoma onset and progression
Source: Cancer Cell Int. 2019 Apr 8;19:89. doi: 10.1186/s12935-019-0804-3 (PMC6454777; doi:10.1186/s12935-019-0804-3)
Supplement: Supplementary file 4 — Additional file 4: Table S2. List of potential tumor suppressor and oncogenic miRNAs. Left; Tumor suppressor miRNAs which are downregulated in HCC and whose higher expressions are associated with good survival of HCC patients from GEO dataset GSE31384 are listed along with hazard ratio (HR), associated p-value and number of patients (N). Right; Oncogenic miRNAs which are upregulated in HCC and whose higher expression are associated with poor survival of HCC patients from GEO dataset GSE31384 are listed along with hazard ratio (HR), associated p-value and number of patients (N). [file 12935_2019_804_MOESM4_ESM.pdf]

Table S2 List of potential tumor suppressor and oncogenic miRNAs. Left; Tumor suppressor miRNAs which are downregulated in HCC and whose higher expressions are associated with good survival of HCC patients from GEO dataset GSE31384 are listed along with hazard ratio (HR), associated p-value and number of patients (N). Right; Oncogenic miRNAs which are upregulated in HCC and whose higher expression are associated with poor survival of HCC patients from GEO dataset GSE31384 are listed along with hazard ratio (HR), associated p-value and number of patients (N).

TS miRNA	HR	p-value	N
**hsa-miR-126-3p**	0.35	1.69E-02	166
**hsa-miR-139-5p**	0.37	4.07E-05	166
**hsa-miR-145-3p**	0.38	7.67E-05	166
**hsa-miR-100-3p**	0.39	7.07E-05	166
**hsa-miR-26a-2-3p**	0.39	1.17E-04	166
**hsa-miR-181c-3p**	0.40	2.24E-04	166
**hsa-miR-149-3p**	0.46	1.11E-03	166
**hsa-miR-99b-3p**	0.46	1.27E-03	166
**hsa-miR-29c-3p**	0.46	1.35E-03	166
**hsa-miR-105-5p**	0.46	1.69E-03	166
**hsa-miR-31-5p**	0.47	1.90E-03	166
**hsa-miR-141-5p**	0.48	2.39E-03	166
**hsa-miR-377-5p**	0.48	2.67E-03	166
**hsa-miR-34b-3p**	0.49	2.66E-03	166
**hsa-miR-208b-3p**	0.49	3.13E-03	166
**hsa-miR-125b-2-3p**	0.50	4.09E-03	166
**hsa-miR-422a**	0.50	4.66E-03	166
**hsa-miR-424-3p**	0.51	4.17E-03	166
**hsa-miR-98-5p**	0.51	4.90E-03	166
**hsa-miR-146b-3p**	0.52	6.63E-03	166
**hsa-miR-302a-5p**	0.52	7.66E-03	166
**hsa-miR-181d-5p**	0.53	6.61E-03	166
**hsa-miR-29a-5p**	0.53	7.54E-03	166
**hsa-miR-10b-5p**	0.55	1.28E-02	166
**hsa-miR-194-3p**	0.55	1.70E-02	166
**hsa-miR-30a-5p**	0.56	1.43E-02	166
**hsa-miR-518a-3p**	0.56	1.54E-02	166
**hsa-miR-183-5p**	0.56	1.80E-02	166
**hsa-miR-29b-3p**	0.57	1.65E-02	166
**hsa-miR-122-3p**	0.57	1.94E-02	166
**hsa-miR-302c-3p**	0.57	2.00E-02	166
**hsa-miR-340-5p**	0.57	2.09E-02	166
**hsa-miR-200b-3p**	0.58	2.33E-02	166
**hsa-miR-496**	0.59	2.59E-02	166
**hsa-miR-219a-1-3p**	0.59	2.64E-02	166
**hsa-miR-378a-3p**	0.59	2.85E-02	166
**hsa-miR-219a-2-3p**	0.59	2.95E-02	166
**hsa-miR-144-3p**	0.60	2.99E-02	166
**hsa-miR-490-5p**	0.60	3.40E-02	166
**hsa-miR-122-5p**	0.60	3.41E-02	166
**hsa-let-7b-3p**	0.60	3.80E-02	166
**hsa-miR-34b-5p**	0.61	3.58E-02	166
**hsa-miR-10b-3p**	0.61	3.68E-02	166
**hsa-miR-135a-5p**	0.61	4.16E-02	166
**hsa-miR-181a-3p**	0.62	4.07E-02	166
**hsa-miR-142-3p**	0.62	4.22E-02	166

OG miRNA	HR	p-value	N
**hsa-miR-938**	2.25	6.33E-04	166
**hsa-miR-628-3p**	2.19	1.02E-03	166
**hsa-miR-675-5p**	2.10	1.94E-03	166
**hsa-miR-551b-3p**	2.05	3.04E-03	166
**hsa-miR-671-3p**	2.03	3.14E-03	166
**hsa-miR-934**	2.03	3.15E-03	166
**hsa-miR-671-5p**	2.02	3.09E-03	166
**hsa-miR-939-5p**	1.94	5.56E-03	166
**hsa-miR-589-3p**	1.93	6.59E-03	166
**hsa-miR-658**	1.91	7.27E-03	166
**hsa-miR-652-3p**	1.90	7.38E-03	166
**hsa-miR-636**	1.90	8.51E-03	166
**hsa-miR-770-5p**	1.88	7.65E-03	166
**hsa-miR-330-3p**	1.88	9.35E-03	166
**hsa-miR-933**	1.84	1.06E-02	166
**hsa-miR-92b-5p**	1.83	1.04E-02	166
**hsa-miR-92b-3p**	1.79	1.35E-02	166
**hsa-miR-877-5p**	1.77	1.59E-02	166
**hsa-miR-374a-5p**	1.75	1.98E-02	166
**hsa-miR-611**	1.71	2.39E-02	166
**hsa-miR-629-3p**	1.71	2.46E-02	166
**hsa-miR-9-3p**	1.69	2.69E-02	166
**hsa-miR-769-3p**	1.66	3.25E-02	166
**hsa-miR-410-3p**	1.66	3.57E-02	166
**hsa-miR-186-5p**	1.63	3.78E-02	166
**hsa-miR-518d-3p**	1.62	4.37E-02	166
**hsa-miR-9-5p**	1.61	4.41E-02	166
