# Supplementary material for: Systems biology based meth-miRNA–mRNA regulatory network identifies metabolic imbalance and hyperactive cell cycle signaling involved in hepatocellular carcinoma onset and progression
Source: Cancer Cell Int. 2019 Apr 8;19:89. doi: 10.1186/s12935-019-0804-3 (PMC6454777; doi:10.1186/s12935-019-0804-3)
Supplement: Supplementary file 5 — Additional file 5: Table S3. List of targets of tumor suppressor and oncogenic miRNAs in meth-miRNA–mRNA network. [file 12935_2019_804_MOESM5_ESM.pdf]

Table S3 List of targets of tumor suppressor and oncogenic miRNAs in meth-miRNA-mRNA network.

Targets of Tumor suppressor miRNAs				
**miR-105-5p**	**miR-149-3p**	**miR-194-3p**	**miR-200b-3p**	**miR-340-5p**
AGFG1	AGFG1	AATF	AGFG1	AGFG1
AGPS	AGPS	AGFG1	AGPS	AGPS
ANAPC7	APEX2	AGPS	ANLN	ANLN
ARF4	ARF4	ANAPC7	APAF1	APAF1
ATP7A	ARL8B	ANLN	ARL8B	ARF4
BUB3	ATP7A	ARF4	ATP7A	ARL8B
CCNA2	AURKA	ARL8B	CCNA2	ATP7A
CCNB1	AURKB	ATP7A	CCT4	BUB3
CCT4	BIRC5	AURKB	CDC73	CCNA2
CCT6A	CAD	BIRC5	CDK2	CCNB1
CDC23	CDC23	BUB3	CEP55	CCT7
CDC73	CDC25A	CCNB1	CKS2	CDC123
CDCA2	CDC7	CCNF	DDX1	CDC23
CENPE	CDCA3	CCT6A	DYNC1LI1	CDC7
CENPH	CDCA8	CCT7	E2F6	CDC73
CEP250	CENPA	CDC23	FAM83D	CDCA3
CEP55	CENPE	CDC25A	FASTKD3	CDK2
CHAF1B	CENPJ	CDC25C	FUBP1	CENPH
CHEK1	CEP250	CDC6	GMPS	CENPJ
CKAP5	CEP55	CDC73	GTPBP4	CHEK1
CPSF6	CHEK1	CDCA8	HDAC2	CKAP5
DARS2	CPSF6	CENPE	KIF11	CPSF6
DCK	CSNK2A1	CENPJ	KIF14	CSNK2A1
DCTN2	DARS2	CEP250	MSH2	DARS2
DDX1	E2F6	CEP55	NCL	DDX1
DNM1L	E2F7	CHEK1	NDC80	E2F6
DYNC1LI1	EFTUD2	CSTF2	NEDD1	E2F7
E2F6	EIF2B1	DCK	NUP43	E2F8
EFTUD2	EIF2S3	DNM1L	POLQ	EXO1
FAM83D	EIF4A3	DYNC1LI1	PPAT	FASTKD3
FIGNL1	EXOSC2	E2F6	PRKAA2	FIGNL1
GMPS	FAM83D	E2F7	RAB23	GMPS
GRPEL2	FANCI	EIF2S3	RAP2A	GRPEL2
HDAC2	GTPBP2	EXO1	RBM12B	HDAC1
ILF2	HDAC1	FAM83D	RIT1	HDAC2
KIF14	HDAC2	FANCI	SART3	HSPA14
KIF2A	HSPA14	FIGNL1	SNRPB2	ISY1
KIFC1	IARS	FUBP1	SNRPD1	KIF11
LARP4B	ISY1	GRPEL2	SNRPE	KIF14
LIN9	KIF11	HDAC1	SPC25	KIF20B
MAD2L1	KIF2C	HDAC2	SQLE	KIF2A
MELK	LARP4B	HJURP	SSB	LARP4B
MKI67	MASTL	HNRNPM	TIMELESS	LIN9
NCAPG2	MCM2	HNRNPR	TRIP13	MAD2L1
NCL	MCM5	ILF2	VRK2	MELK
NEDD1	MKI67	ISY1	YWHAQ	MSH2
NEIL3	MYO19	KIF11		NAE1
NUP43	NCAPG2	KIF14		NCAPG2
PA2G4	NKIRAS2	KIF20A		NEDD1
POLA2	NME7	KIF2A		NKIRAS2
POLE2	NUP43	KIF2C		NME7

Targets of Tumor suppressor miRNAs				
**miR-105-5p**	**miR-149-3p**	**miR-194-3p**	**miR-200b-3p**	**miR-340-5p**
PPAT	PA2G4	LARP4B		NUF2
PPIL1	PLK1	LIN9		PCNA
PRKAA2	POLA2	MAPKAPK5		PLK1
PSMD1	POLQ	MCM2		POLQ
PSMD14	POLR2H	MCM5		POLR2H
PTTG1	POLR3F	ME2		PPAT
RAP2A	PPIL1	MKI67		PRKAA2
RBM12B	PPM1G	MSH2		PUS7
RHEB	PRC1	NARS		RAB10
RIT1	PRKAA2	NAT10		RAB23
SEH1L	PRKAG1	NCAPG2		RAD51AP1
SLC4A1AP	PUS7	NCBP2		RAD54L
SPC25	RAB10	NCL		RAP2A
SQLE	RHEB	NME6		RBM12B
TDG	RIT1	NME7		RIOK1
TIMELESS	SEH1L	NUF2		RIT1
TTK	SF3B1	NUP37		SEH1L
	SNRPB2	PA2G4		SF3B1
	SPC25	PCNA		SSB
	TIMELESS	POLA2		TDG
	TK1	POLQ		TIPIN
	UBE2O	POLR2H		TOP2A
	WNK1	PPAT		VRK1
	YWHAQ	PPIH		VRK2
	ZNF346	PPIL1		WNK1
		PRKAA2		XPOT
		PSMA1		YWHAQ
		PSMB5		
		PUS7		
		RAB10		
		RAB23		
		RAD51AP1		
		RAP2A		
		RHEB		
		RIT1		
		RNASEH1		
		SF3B4		
		SNRPD1		
		SNRPE		
		SPC25		
		STMN1		
		TIMELESS		
		TK1		
		TPR		
		TRIP13		
		TTK		
		UBE2O		
		WNK1		
		XPOT		
		YWHAQ		
		ZNF346		

Targets of oncogenic miRNAs				
**miR-330-3p**	**miR-671-3p**	**miR-671-5p**	**miR-877-5p**	**miR-939-5p**
ADHFE1	ACSM2A	ACSM2A	ADH1A	ACADS
ALDH2	ADH6	ACSM5	ADH1C	ACSM5
ALPL	AGXT	ADH6	ALDH9A1	ADH4
CBR4	AKR1D1	AGXT	ALPL	ADHFE1
CFP	AMDHD1	AMDHD1	C1RL	AGXT
CSAD	C1RL	C1RL	CYP2C19	AKR1D1
CYB5D2	C7	C7	CYP2C8	ALDH2
CYP2C9	CAT	CAT	CYP2C9	ALDH8A1
CYP3A43	CFP	CBS	CYP3A43	ALPL
CYP4F12	COLEC10	CFP	CYP4A11	C1RL
CYP8B1	CYP2C9	CYB5D2	CYP4F12	CBR4
DBT	CYP2E1	CYP2E1	CYP8B1	CNDP1
DHTKD1	CYP4F2	CYP4V2	DAO	CSAD
DMGDH	CYP4F3	CYP8B1	DBT	CYP27A1
ETFDH	CYP4V2	DBH	DHRS1	CYP2C8
F12	CYP8B1	DHRS1	DHTKD1	CYP2E1
FMO2	DBT	DHTKD1	EPHX2	CYP3A43
GBA3	DHRS1	DPYS	F7	CYP4A11
GFOD1	DHTKD1	FDX1	F9	CYP4F12
GGT5	FDX1	FMO2	FBP1	CYP4V2
GPT	FMO2	FMO3	FGB	CYP8B1
HABP2	FMO3	GAMT	FMO2	DAO
HGD	GCDH	GCDH	FMO3	DBH
INMT	GFOD1	GFOD1	FTCD	DBT
MASP1	GGT5	GPT2	GAMT	DHTKD1
MSRA	GPT2	GZMH	HAAO	DPYS
PCK1	HAAO	HAAO	HAO2	EPHX2
PHYHD1	HABP2	HABP2	IVD	F12
PPARGC1A	HEBP1	HADH	KLKB1	F7
SLC25A11	HMGCS2	HMGCS2	MASP1	FMO2
STEAP4	HPN	HPN	MST1	FTCD
TK2	INMT	INMT	NDST3	GFOD1
UPB1	LDHD	IVD	PCK2	GNMT
UROC1	MAN1C1	LDHD	PHYHD1	GOT2
	MST1	MAN1C1	QDPR	GPT2
	NDST3	MSRA	STEAP4	GZMH
	PCK2	MST1	TMPRSS6	HAAO
	PHYHD1	NDST3	XDH	HABP2
	SLC25A11	OGDHL		HAO1
	TAT	PCK2		HAO2
	TK2	PHYHD1		HEBP1
	TMPRSS6	RDH16		HPD
	UPB1	STEAP4		IVD
	XDH	TAT		KLKB1
		TK2		LDHD

Targets of oncogenic miRNAs				
**miR-330-3p**	**miR-671-3p**	**miR-671-5p**	**miR-877-5p**	**miR-939-5p**
		UPB1		MASP1
		XDH		MSRA
				MST1
				NDST3
				PCK1
				PCK2
				PHYHD1
				PIPOX
				PPARGC1A
				SLC25A11
				TAT
				TK2
				TMPRSS6
				UPB1
				UROC1
				XDH
