# Supplementary material for: Systems biology based meth-miRNA–mRNA regulatory network identifies metabolic imbalance and hyperactive cell cycle signaling involved in hepatocellular carcinoma onset and progression
Source: Cancer Cell Int. 2019 Apr 8;19:89. doi: 10.1186/s12935-019-0804-3 (PMC6454777; doi:10.1186/s12935-019-0804-3)
Supplement: Supplementary file 6 — Additional file 6: Table S4. List of hypermethylated tumor suppressors and hypomethylated oncogenes in meth-miRNA–mRNA network. [file 12935_2019_804_MOESM6_ESM.pdf]

Table S4 List of hypermethylated tumor suppressors and hypomethylated oncogenes in meth-miRNA-mRNA network.

**Hypermethylated tumor suppressors**					
ACADS	C5	CYP4F12	FCN2	KLKB1	SLC25A11
ACSM5	C7	CYP4F3	FDX1	LDHD	SLC27A5
ADH1A	CAT	CYP4V2	FGB	MASP1	SRD5A2
ADH6	CBR4	CYP8B1	FMO2	MSRA	STEAP4
ADI1	CBS	DAO	FMO3	NDST3	TAT
AGXT	CFB	DBH	GFOD1	NR1I2	TK2
AKR1D1	CFP	DGAT2	GGT5	OGDHL	TMPRSS6
ALAS1	CSAD	DHTKD1	GOT2	PHYHD1	UROC1
ALDH2	CYB5D2	ECHS1	GPT	PROZ	
ALDH9A1	CYP2C19	EPHX2	HEBP1	QDPR	
ALPL	CYP2C8	ETFDH	HGFAC	RDH16	
AMDHD1	CYP2C9	F12	HPD	SAT2	
C1RL	CYP3A43	F2	INMT	SDS	
**Hypomethylated oncogenes**					
AATF	CDCA3	EIF2S2	MAD2L1	POLA2	SGOL2
AGFG1	CDK2	EIF2S3	MASTL	POLR3C	SNRPA
ANLN	CDK5	EXO1	MCM2	POLR3F	SNRPD1
APAF1	CENPH	FAM83D	ME2	PPAT	SPC25
APEX2	CENPJ	FASTKD3	MELK	PPM1G	SQLE
ATP7A	CEP250	FEN1	MKI67	PRKAA2	SSB
AURKB	CHAF1B	FIGNL1	MSH2	PRKDC	STMN1
BIRC5	CHEK1	GRPEL2	NAE1	PSMA1	TDG
BUB3	CKAP5	GTPBP4	NCAPG	PTTG1	TK1
CAD	CSNK2A1	HDAC1	NCL	RFC4	TOP2A
CCNB1	CSTF2	ILF2	NME7	RIT1	UBE2O
CCNF	DARS2	KIF11	NUP43	RNASEH1	UBE2T
CCT4	DLGAP5	KIF14	OIP5	RNASEH2A	VRK2
CCT6A	DNM1L	KIF15	PBK	RRM2	
CDC123	E2F7	LARP4B	PCNA	SF3B4	
CDC20	EFTUD2	LIN9	PLK1	SGOL1	
