# Supplementary material for: Systems biology based meth-miRNA–mRNA regulatory network identifies metabolic imbalance and hyperactive cell cycle signaling involved in hepatocellular carcinoma onset and progression
Source: Cancer Cell Int. 2019 Apr 8;19:89. doi: 10.1186/s12935-019-0804-3 (PMC6454777; doi:10.1186/s12935-019-0804-3)
Supplement: Supplementary file 7 — Additional file 7: Fig. S3. Hypermethylation driven inhibition of metabolic pathways and hypomethylation driven activation of cell cycle signaling are associated with aggressive disease state in HCC. Dot-plot showing meth-GS scores of HCC patients who experienced or did not experience vascular invasion in TCGA database (a). Dot-plot showing meth-GS scores of HCC patients representing different tumor stages (stage I, II or III) in TCGA database (b). Dot-plot showing meth-GS scores of HCC patients representing different tumor grades (G1, G2 or G3) in TCGA database (c). Dot-plot showing meth-GS scores of HCC patients representing different pathological tumor size (T1, T2 or T3) in TCGA database (d). *P < 0.05; **P < 0.01; ns, not significant, Student’s t-test. [file 12935_2019_804_MOESM7_ESM.pdf]

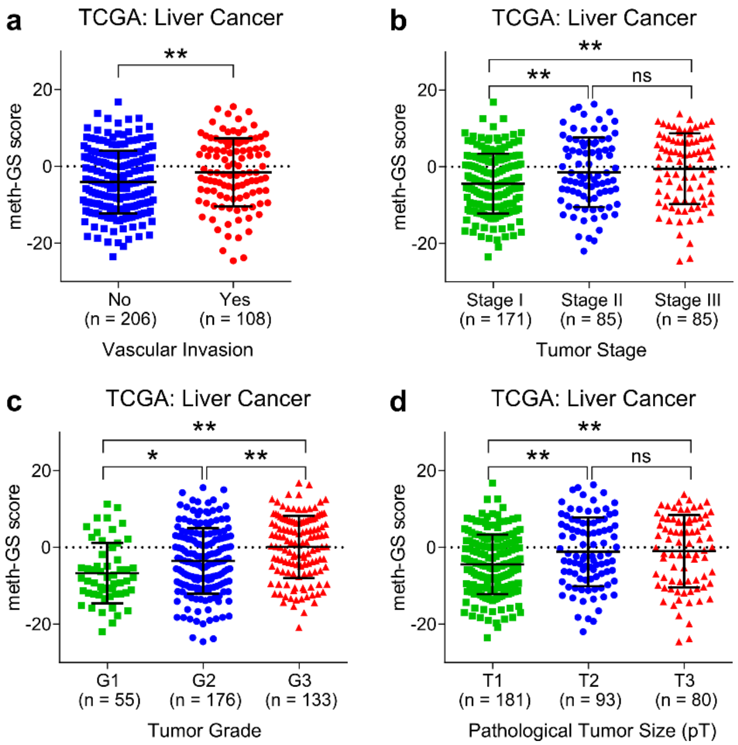

Fig. S3 Hypermethylation driven inhibition of metabolic pathways and hypomethylation driven activation of cell cycle signaling are associated with aggressive disease state in HCC. Dot-plot showing meth-GS scores of HCC patients who experienced or did not experience vascular invasion in TCGA database (**a**). Dot-plot showing meth-GS scores of HCC patients representing different tumor stages (stage I, II or III) in TCGA database (**b**). Dot-plot showing meth-GS scores of HCC patients representing different tumor grades (G1, G2 or G3) in TCGA database (**c**). Dot-plot showing meth-GS scores of HCC patients representing different pathological tumor size (T1, T2 or T3) in TCGA database (**d**). *P <0.05; **P < 0.01; ns, not significant, Student's t-test.
